# Prox1 Regulates the Notch1-Mediated Inhibition of Neurogenesis

**DOI:** 10.1371/journal.pbio.1000565

**Published:** 2010-12-21

**Authors:** Valeria Kaltezioti, Georgia Kouroupi, Maria Oikonomaki, Evangelia Mantouvalou, Athanasios Stergiopoulos, Aristidis Charonis, Hermann Rohrer, Rebecca Matsas, Panagiotis K. Politis

**Affiliations:** 1 Center for Basic Research, Biomedical Research Foundation of the Academy of Athens, Athens, Greece; 2 Laboratory of Cellular and Molecular Neurobiology, Hellenic Pasteur Institute, Athens, Greece; 3 Department of Neurochemistry, Max-Planck Institute for Brain Research, Frankfurt/Main, Germany; Stanford University, United States of America

## Abstract

Activation of Notch1 signaling in neural progenitor cells (NPCs) induces self-renewal and inhibits neurogenesis. Upon neuronal differentiation, NPCs overcome this inhibition, express proneural genes to induce Notch ligands, and activate Notch1 in neighboring NPCs. The molecular mechanism that coordinates Notch1 inactivation with initiation of neurogenesis remains elusive. Here, we provide evidence that Prox1, a transcription repressor and downstream target of proneural genes, counteracts Notch1 signaling via direct suppression of *Notch1* gene expression. By expression studies in the developing spinal cord of chick and mouse embryo, we showed that Prox1 is limited to neuronal precursors residing between the Notch1+ NPCs and post-mitotic neurons. Physiological levels of Prox1 in this tissue are sufficient to allow binding at *Notch1* promoter and they are critical for proper *Notch1* transcriptional regulation in vivo. Gain-of-function studies in the chick neural tube and mouse NPCs suggest that Prox1-mediated suppression of *Notch1* relieves its inhibition on neurogenesis and allows NPCs to exit the cell cycle and differentiate. Moreover, loss-of-function in the chick neural tube shows that Prox1 is necessary for suppression of *Notch1* outside the ventricular zone, inhibition of active Notch signaling, down-regulation of NPC markers, and completion of neuronal differentiation program. Together these data suggest that Prox1 inhibits *Notch1* gene expression to control the balance between NPC self-renewal and neuronal differentiation.

## Abstract 2

During development of the spinal cord, Prox1 controls the balance between proliferation and differentiation of neural progenitor cells via suppression of *Notch1* gene expression.

## Author Summary

Early during development, neural progenitor cells (NPCs) can either proliferate or differentiate into neurons. Thus, generation of the correct number of neurons is governed by a tightly regulated balance between proliferation and differentiation, and disruption of this balance can result in severe developmental deficits, malformations, or cancers. Notch1 is a member of the Notch family of receptors, which make up a highly conserved cell signaling system. Notch1 signaling has been shown to inhibit NPC differentiation and to promote self-renewal, thereby allowing NPCs to divide and progressively generate the enormous number of neurons present in the central nervous system. The molecular mechanism by which NPCs overcome Notch1-mediated inhibition in order to differentiate into neurons, however, is not completely understood. In this study, we show that Prox1, a homeobox transcriptional repressor, plays a fundamental role in the switch to differentiation by suppressing the expression of Notch1 receptor, thereby preventing newly produced neuronal precursors from receiving inhibitory signals from Notch ligands present in neighboring cells. This transcriptional repression may regulate cell cycle exit and differentiation of NPCs as they migrate towards different regions and adopt their final cell fates. We suggest that Prox1 may exert its known influence on embryonic development, organ morphogenesis, and cancer through its ability to counteract Notch1 signaling.

## Introduction

Notch1 signaling plays an important role in the maintenance of NPCs in the undifferentiated state by inhibiting neuronal differentiation [1]–[3]. In the developing nervous system, NPCs initially undergo symmetric divisions to expand the available pool of progenitor cells, while at later stages, during the neurogenic phase, they switch to asymmetric divisions generating one progenitor cell and one neuronal precursor destined to become a neuron. In these nascent neurons, proneural genes induce the expression of Notch ligands such as Delta1 and Jagged, which in turn activate Notch1 in neighboring NPCs. The interaction of Notch1 with its ligands results in cleavage of the Notch intracellular domain (NICD) by the presenilin/γ-secretase complex [4] and its translocation to the nucleus where it forms a complex with RBP-J (CBF1). This complex activates transcription of the basic helix-loop-helix (bHLH) genes *Hes1* and *Hes5*, which act as downstream effectors of Notch1 to down-regulate proneural gene expression and inhibit neurogenesis [5]–[9]. By this process, also known as lateral inhibition, newly produced neurons are thought to feedback and inhibit neighboring NPCs from differentiating, thus regulating the number of neurons born at a given time and maintaining a pool of NPCs for generation of subsequent neurons and glia. However, in these asymmetrically produced precursors that have been instructed to become post-mitotic neurons, active Notch1 signaling has to be terminated [2],[10]. Although the reduced expression of Notch1 ligands by neighboring cells contributes to a decrease in Notch1 signaling, additional mechanisms should operate to protect nascent neuronal precursors against Notch1 signals from neighboring cells and maintain neuronal fate and specification. In vertebrate CNS the molecular mechanism that inactivates Notch1 signaling in cells destined to become neurons during the initial phases of differentiation remains largely unknown [10].

To this end, we hypothesized that proneural genes, which are expressed into the Notch1 positive area in the neural tube [5], may activate downstream effector genes that will coordinate cell-autonomously the induction of neuronal differentiation with the inhibition of Notch1 signaling. In this regard two possible mechanisms, non-mutually exclusive, may be envisaged: either inactivation of key component(s) in the Notch1 signaling axis via protein-protein interactions and/or degradation, or down-regulation by transcriptional repression. Detailed expression studies have previously shown that newly-born neurons readily down-regulate *Notch1* expression at the mRNA level so that cells that exit the ventricular zone (VZ) rapidly suppress *Notch1* gene expression [11]–[13]. This observation indicates that a potential mechanism, operating in neuronal precursors to desensitize them from responding to Delta/Jagged signal-sending cells, is to down-regulate the expression of the Notch1 itself by transcriptional repression.

Prox1, a homeobox transcription repressor, acting downstream of proneural genes during neurogenesis, is a good candidate for such a function. Interestingly, *Prospero*, the *Drosophila* homologue of Prox1 in vertebrates, is a critical regulator of the balance between self-renewal and differentiation in neural stem cells (NSCs) [14],[15]. Prospero suppresses the genetic program for self-renewal of NSCs and cell cycle progression, while it activates genes for terminal neuronal differentiation [15],[16]. Neuroblasts that lack Prospero form tumors in the embryonic nervous system of *Drosophila* [15]. Notch signaling appears to have the opposite function by promoting self-renewal and neuroblast identity [17]–[19]. Most important, *Prox1* in vertebrates is expressed in the boundaries between Notch1+ and Notch1− cells during development of the spinal cord and is transiently expressed in neuronal precursors but not in terminally differentiated neurons in this region [20]–[22]. Multiple lines of evidence suggest important roles for Prox1 in different aspects of embryonic development and morphogenesis, while mouse embryos deficient in *Prox1* die at E14.5, a critical time point in the development of many different organs [23],[24]. Thus, *Prox1* has been previously shown to have essential roles during lymphatic, hepatocyte, pancreatic, heart, lens, retinal, and spinal cord development [20],[23],[25]–[29]. Collectively, these observations indicate that *Prox1* is involved in many key developmental decisions during organ morphogenesis.

Here we provide functional evidence that Prox1 is directly implicated in *Notch1* gene suppression during neurogenesis in the developing spinal cord and thus co-ordinately regulates Notch1 signaling inactivation with neuronal differentiation. In particular, we showed that physiological levels of endogenous Prox1 are sufficient to allow binding at the *Notch1* promoter locus in vivo, and gain-and-loss-of-function studies suggest that Prox1 levels are essential for proper regulation of the endogenous *Notch1* gene in vivo. Moreover, these studies indicate that Prox1-mediated *Notch1* suppression controls cell cycle exit and differentiation of NPCs. Together our data imply that Prox1 is involved in the transition of NPCs from self-renewal to neuronal differentiation via direct suppression of *Notch1*.

## Results

### *Prox1* Is Expressed in an Intermediate Zone between *Notch1*+ Cells and Post-Mitotic Neurons in the Early Spinal Cord

**Figure 1:**
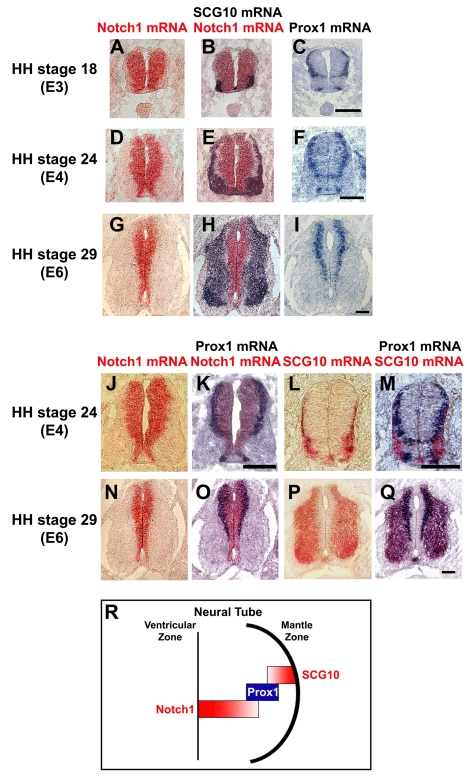
Comparison of spatiotemporal expression patterns of *Prox1*, *Notch1*, and *SCG10* genes in early embryonic chick spinal cord. (A–I) Double in situ hybridizations with *Notch1* (red) and *SCG10* (blue) on sections from HH stages 18 (A–B), 24 (D–E), and 29 (G–H), and comparison with *Prox1* (C, F, and I) single in situ hybridizations on adjacent sections. The sections were from the thoracic level of spinal cord. (J–Q) Double in situ hybridizations with *Notch1* (red) and *Prox1* (blue) on sections from HH stages 24 (J–K, thoracic level) and 29 (N–O, thoracic level), and double in situ hybridizations with *SCG10* (red) and *Prox1* (blue) on sections from HH stages 24 (L–M, cervical level) and 29 (P–Q, thoracic level). Scale bars: 100 µm. (R) Schematic representation of the *Prox1* expression pattern in neural tube during early embryonic development.

There is an inverse spatiotemporal correlation between expression of *Notch1* and neuronal markers such as *SCG10*, in a way that differentiated neurons expressing *SCG10* appear later in development and localize in the mantle zone (MZ), whereas *Notch1* and *Hes5* are highly expressed early in development while later reduced and detected in NPCs of the VZ (Figure 1A, 1B, 1D, 1E, 1G, 1H and unpublished data) [11]–[13]. Moreover, an intermediate zone of cells, negative for both markers, is also evident. These observations suggest that prior to acquisition of a terminally differentiated neuronal character the expression of *Notch1* mRNA has to be strongly down-regulated. However, the molecular mechanism that actively directs suppression of *Notch1* expression to relieve its inhibitory action on neurogenesis remains totally unknown. As Prox1 acts downstream of proneural genes and given the inverse correlation between proneural genes and active Notch1 signaling, we considered Prox1 as a potential factor that could be involved in this suppression. Comparative analysis of *Prox1*, *Notch1*, and *SCG10* expression by single and double in situ hybridization revealed that *Prox1* is expressed in the intermediate zone of cells between *Notch1*+ NPCs and *SCG10*+ post-mitotic neurons (Figure 1 and Figure S1). Most important, *Prox1* positive signal is observed in a cell population that laminates and demarcates the area of Notch1+ cells, both in the chick and mouse embryonic spinal cord (Figure 1J, 1K, 1N, 1O and Figure S1Q–S1T), probably marking a transitory cell population in the medio-lateral axis, that migrates towards the MZ to differentiate into post-mitotic neurons (Figure 1R). This expression pattern is consistent with a role in suppressing *Notch1* expression at the mRNA level.

### Prox1 Is Directly Involved in *Notch1* Gene Suppression

**Figure 2:**
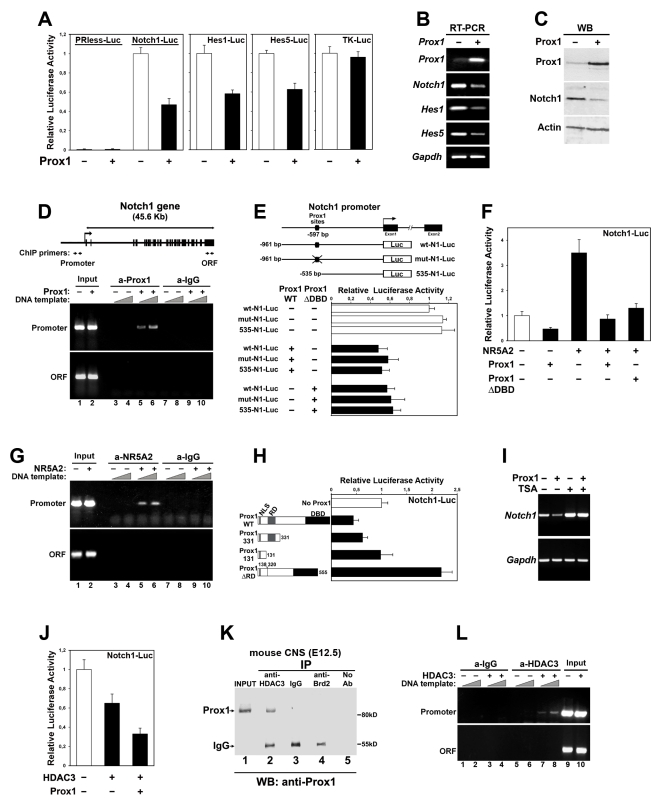
Prox1-mediated transcriptional repression of the *Notch1* gene promoter in N2A cells is facilitated by NR5A2 and HDAC3. (A) Transcriptional assays in N2A co-transfected with a *Prox1* expression plasmid and luciferase reporter constructs containing human *Notch1*, mouse *Hes1*, mouse *Hes5*, or human *thymidine kinase* promoters, as well as empty vector (PRless). In all luciferase experiments shown in this figure, data are represented as the mean ± SD of quadruplicate assays. In all cases *p*<0.01, except TK-Luc and PRless, *p*>0.1. (B–C) RT-PCR (B) and Western blot (C) analysis of Prox1 overexpression in N2A, for *Prox1*, *Notch1*, *Hes1*, *Hes5*, and *Gapdh* genes (B), and Prox1, Notch1, and Actin proteins (C), as indicated. (D) ChIP analysis of the binding of Prox1 to *Notch1* promoter in N2A transiently transfected with a Prox1 construct or a control empty vector. The organization of the *Notch1* gene is schematically represented in the top panel. Exons are represented as black boxes. The primer pairs used to amplify the corresponding DNA sequences are indicated with arrows below the schematic drawing. (E) Transcriptional assays in N2A co-transfected with WT Prox1, ΔDBD Prox1, or empty vector, and WT Notch1-luc, mut-Notch1-Luc, or 535-Notch1-Luc reporter constructs. Schematic of the *Notch1* promoter and corresponding mutations are indicated in the top of the panel. In all cases *p*<0.01. (F) Transcriptional assays in N2A co-transfected with WT Notch1-Luc construct and various combinations of expression vectors, as indicated. *p*<0.01 for NR5A2 alone versus NR5A2/Prox1, and NR5A2 alone versus NR5A2/ΔDBD-Prox1. (G) ChIP analysis of the binding of NR5A2 to *Notch1* promoter in N2A cells transfected with a NR5A2 construct or a control empty vector. (H) Transcriptional assays in N2A co-transfected with Notch1-Luc construct and various deletion constructs for Prox1. Schematic of the Prox1 deletion constructs is presented in the left of the panel. Grey box indicates the RD domain, and black box indicates the DBD domain. In all cases *p*<0.01, except 331-Prox1 versus No-Prox1, *p*<0.05; 131-Prox1 versus No-Prox1, *p*>0.1. (I) RT-PCR analysis of Prox1 overexpression in N2A, treated with 150 nM TSA or vehicle alone, for endogenous *Notch1* and *Gapdh* genes, as indicated. (J) Transcriptional assays in N2A co-transfected with Notch1-Luc construct and *HDAC3* expression vector in the presence or absence of Prox1. *p*<0.05 for WT versus HDAC3; *p*<0.01 for WT versus HDAC3+Prox1; *p*<0.05 for HDAC alone versus HDAC3+Prox1. (K) Prox1 binds HDAC3 in vivo. Cell lysates from mouse embryonic CNS (E12.5) were subjected to immunoprecipitations with anti-HDAC3 antibody, control anti-rabbit IgGs, or control anti-Brd2 antibody, followed by immunoblotting with anti-Prox1 antibody. The positions of Prox1 and heavy chain of IgGs are shown on the left. (L) ChIP analysis of the binding of HDAC3 to *Notch1* promoter in N2A transfected with a HDAC3 construct or a control empty vector.

To initially test this hypothesis, we performed transcriptional assays in mouse neuroblastoma Neuro2A cells (N2A). We first used previously published promoter-luciferase constructs for mammalian *Notch1* [30] or *Hes1* and *Hes5* genes [31],[32] to evaluate the effect of Prox1 on transcription regulation. After transient transfections of N2A with a mouse (Figure 2A) or human (Figure S2A) *Prox1* expression vector, the activity of *Notch1* promoter was significantly decreased, whereas a control construct carrying the human *Thymidine-kinase* (TK) promoter remained unaffected. Prox1 was also able to suppress the activities of *Hes1* and *Hes5* promoters, presumably via its action on *Notch1* (Figure 2A). To determine whether Prox1 affects in a similar manner the expression of endogenous genes, we stably overexpressed *Prox1* in N2A. These cells showed reduced levels of *Notch1* mRNA and protein levels, as well as *Hes1* and *Hes5* mRNA levels (Figure 2B and 2C), indicative of a significant repression in Notch signaling. We next asked whether Prox1 directly interacts with the chromatin of the proximal *Notch1* gene promoter by performing chromatin immunoprecipitation (ChIP) experiments (Figure 2D). An antibody to Prox1 co-precipitated the proximal *Notch1* promoter sequence in chromatin prepared from Prox1-transfected cells, but not from mock-transfected N2A cells. In addition, sequences from the distal 3′-end of the *Notch1* gene were not precipitated with this antibody. Similarly, control IgGs were not able to precipitate the proximal *Notch1* promoter sequence, further suggesting a direct interaction between Prox1 and chromatin from *Notch1* promoter.

**Figure 3:**
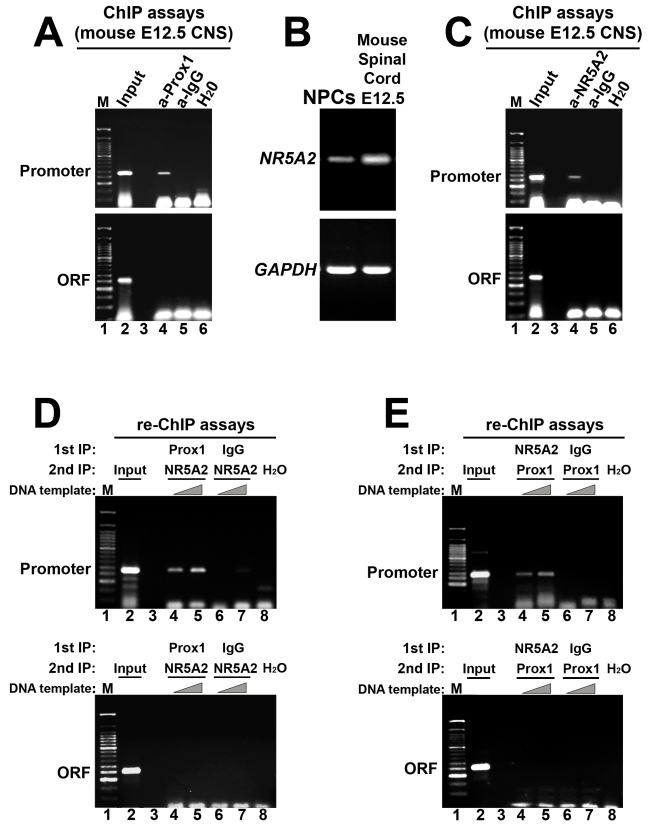
Prox1 and NR5A2 interact in vivo with the promoter of *Notch1* gene. (A) ChIP analysis of the binding of Prox1 to proximal *Notch1* promoter in chromatin prepared from the CNS of E12.5 mouse embryos. The primer pairs used to amplify the corresponding DNA sequences are indicated with arrows below the schematic drawing in Figure 2D. (B) RT-PCR analysis in NPCs cultured in vitro, and E12.5 mouse spinal cords, for the detection of *NR5A2* and *Gapdh* mRNAs, as indicated. (C) ChIP analysis of the binding of NR5A2 to proximal *Notch1* promoter in chromatin prepared from the CNS of E12.5 mouse embryos. (D–E) Re-ChIP experiments were performed in the CNS of E12.5 mouse embryos on the *Notch1* promoter (top panels) and compared with the 3′ *Notch1* coding sequence (ORF, lower panels) using anti-Prox1, anti-NR5A2, or control antibodies in a serial manner. The antibodies used in each round of precipitation are indicated in the top of each panel. The primer pairs used to amplify the corresponding DNA sequences are indicated with arrows below the schematic drawing in Figure 2D.

To better understand the suppressive function of *Prox1* on *Notch1* promoter, different mutants of both *Notch1* promoter construct and Prox1 protein were constructed and utilized in luciferase assays. We first identified on Notch1 promoter two overlapping consensus binding sites for Prox1 [33], conserved between human, mouse, and rat, located 597 bp upstream of the translation start site. However, by mutating both of these sites (mut-N1-Luc) or deleting a 426 bp sequence containing these sites (535-N1-Luc), we observed that Prox1 is still sufficient to suppress Notch1 promoter activity (Figure 2E). Furthermore, deletion of the DNA binding domain (DBD) from the carboxy terminal of Prox1 protein was not sufficient to relieve the Prox1-mediated suppression in *Notch1* promoter activity (Figure 2E and Figure S2B), indicating that Prox1 acts on *Notch1* as transcriptional co-repressor via another factor. There are two previously reported transcription factors that utilize Prox1 as co-repressor via direct interactions to suppress gene expression in other systems, NR5A2 (Nuclear Receptor 5A2) and SF1 (Steroidogenic Factor 1), both belonging to the family of orphan nuclear receptors [34]–[36]. NR5A2 is able to activate Notch1-luc construct in N2A cells (Figure S2D) and is expressed in the mammalian CNS (Figure 3B and Figure S3) [37]. Most important, both wt Prox1 and ΔDBD-Prox1 are sufficient to suppress the NR5A2-mediated induction of Notch1-luc construct (Figure 2F). By performing ChIP experiments with the NR5A2 antibody on NR5A2-transfected and mock-transfected N2A cells, we showed that NR5A2 specifically binds to the proximal *Notch1* promoter sequence (Figure 2G), indicating that Prox1 is likely to be recruited on the *Notch1* promoter to suppress its activity via direct interactions with NR5A2.

Moreover, by using a series of deletion mutants for the Prox1 protein in luciferase assays, we were able to map the repressive function in the N-terminal domain (Figure 2H and Figure S2C). It has been shown that Prox1 achieves its repressive action on gene promoters via a previously described domain in the N-terminal end of the protein, known as Repression Domain (RD) [35],[38], which is not involved in the direct interaction with the NR5A2 [35]. Deletion of this domain abolishes the ability of Prox1 to suppress *Notch1* promoter or the NR5A2-mediated induction of Notch1-luc (Figure 2H and Figure S2E), and instead Prox1ΔRD is now able to enhance *Notch1* promoter activity (Figure 2H). It has also been previously reported that the RD domain has a repressive function on transcription by interacting and utilizing the activity of Histone Deacetylase 3 (HDAC3) [35],[38]. Consistently, treatment of N2A cells with an HDAC inhibitor (trichostatin-A, TSA) is sufficient to derepress endogenous *Notch1* gene transcription and Notch1-luc vector activity, as well as to abolish the Prox1-mediated *Notch1* suppression (Figure 2I and Figure S2F and S2G). Moreover, HDAC3 over-expression suppresses *Notch1* promoter activity, which is further aggravated by *Prox1* co-expression (Figure 2J), indicating that HDAC3 synergizes with Prox1 to suppress *Notch1* promoter. We then directly addressed whether Prox1 interacts with HDAC3 in the mouse CNS. First, by performing RT-PCR, Western blot, and immunostainings, we showed that HDAC3 is expressed in mouse embryonic spinal cord and mouse NPCs isolated from the same tissue and cultured in vitro (Figure S4 and unpublished data). In addition, a co-immunoprecipitation assay showed that Prox1 could be co-precipitated with HDAC3 from protein extracts of mouse E12.5 CNS tissue (Figure 2K). Moreover, by performing ChIP experiments with the HDAC3 antibody on HDAC3-transfected and mock-transfected N2A cells, we showed that HDAC3 specifically binds to the proximal *Notch1* promoter sequence (Figure 2L). These data indicate that endogenous Prox1 and HDAC3 proteins indeed form a complex in the developing mouse spinal cord and that Prox1 utilizes HDAC3 inhibitory activity on transcription to suppress *Notch1* gene expression.

### Prox1 and NR5A2 Directly Interact with the Endogenous *Notch1* Promoter In Vivo

We next investigated whether physiological levels of endogenous Prox1 and NR5A2 are sufficient to allow binding at the *Notch1* promoter locus in vivo. Thus, by performing in vivo ChIP experiments, we showed that endogenous Prox1 protein directly interact with the *Notch1* promoter locus in the mouse embryonic CNS. In particular, an antibody to Prox1 co-precipitated the proximal *Notch1* promoter sequence, but not the 3′ *Notch1* coding sequence, in chromatin prepared from E12.5 mouse CNS (Figure 3A). To address the same question for NR5A2, we first showed that this gene is expressed in E12.5 mouse embryonic spinal cord and NPCs isolated from the same tissue and cultured in vitro (Figure 3B and Figure S3). Similar to Prox1 results, an antibody to NR5A2 was sufficient to specifically precipitate the proximal *Notch1* promoter sequence in chromatin prepared from E12.5 mouse CNS (Figure 3C). Therefore we conclude that Prox1 and NR5A2 specifically bind in vivo the *Notch1* gene promoter.

We next tested whether Prox1 and NR5A2 could form a complex on chromatin over the *Notch1* promoter locus by performing sequential ChIP assays (re-ChIP) in the mouse embryonic CNS. In these experiments, Prox1, NR5A2, or control IgG containing chromatin, purified by the first immunoprecipitation, was eluted from the protein A/G beads and subjected to a second immunoprecipitation with antibodies recognizing NR5A2 or Prox1 in a crosswise manner. As shown in Figure 3D, re-ChIP for NR5A2 following an initial ChIP for Prox1 generated specific enrichment of the *Notch1* promoter sequence, but not of the 3′ *Notch1* coding sequence or *Notch1* promoter after initial ChIP with control IgG. Reciprocal re-ChIP assays, in which the order of the antibodies was inverted, generated identical results (Figure 3E). These data together with the observation that Prox1 acts on *Notch1* promoter as a co-repressor imply that the effect of Prox1 on *Notch1* expression could be mediated through NR5A2.

### Prox1 Enhances Neurogenesis and Inhibits Self-Renewal and Astrogliogenesis in Mouse NPCs

**Figure 4:**
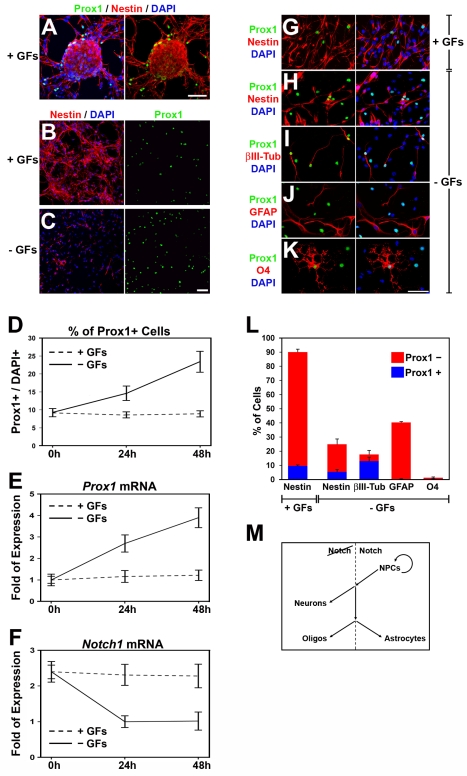
Prox1 is expressed in mouse NPCs cultured in vitro and up-regulated upon differentiation. (A–C) Double Prox1/Nestin immunostainings of NPCs isolated from mouse spinal cords of E14.5 embryos, and cultured in vitro, either as neurospheres (A) or dissociated cells (B–C), in the presence (A–B) or absence of GFs (C). Scale bar: 50 µm. (D) Quantification of Prox1+ cells in NPCs cultured in the presence (dotted line) or absence (solid line) of GFs. The number of Prox1+ cells is expressed as percentage of the total number of DAPI+ cells. (E–F) Relative expression levels of *Prox1* (E) and *Notch1* (F) mRNA in NPCs cultured in the presence (dotted line) or absence (solid line) of GFs, measured with quantitative real time RT-PCR. (G–L) Double immunostainings of NPCs with Prox1 (green) and various markers (red), as indicated, cultured in the presence (G) or absence of GFs (H–K). Scale bar: 50 µm. Quantification of Prox1+ (blue) or Prox1− (red) cells is shown in (L). (M) Schematic representation of the role of active Notch signaling in regulating self-renewal and differentiation of NPCs.

To investigate whether the effect of Prox1 on *Notch1* gene expression is consistent with the function of Notch1 signaling in self-renewal and differentiation of NPCs, we employed an in vitro culture system using NPCs from embryonic mouse spinal cord, which have the ability to self-renew and proliferate when cultured in the presence of growth factors (GFs), as well as to differentiate into neurons, astrocytes, and oligodendrocytes upon withdrawal of GFs [39]. In these cultures, endogenous *Prox1* is up-regulated upon induction of differentiation at the mRNA level and a concomitant induction in the number of Prox1+ cells is observed upon withdrawal of GFs (Figure 4A–4E). In contrast, *Notch1* mRNA levels are reduced and inversely correlated with the induction of *Prox1* upon withdrawal of GFs (Figure 4E and 4F), supporting the repressive action of Prox1 on *Notch1* transcription. Interestingly, 2 d after in vitro differentiation, Prox1 is mainly expressed in the majority of βIII-tubulin+ early differentiating neurons but also in a fraction of nestin+ NPCs that remained undifferentiated (Figure 4G–4L). These Nestin+/Prox1+ cells may represent a transitory cell population of intermediate precursors similar to the transitory cell population observed in the intermediate zone of the chick and mouse spinal cord (Figure 1 and Figure S1) [20],[22]. Moreover, Prox1 is also expressed in a subset of oligodendrocytes, although the numbers of these cells are generally low in this experimental system (Figure 4K and 4L). Most important, Prox1 is excluded from GFAP+ astrocytes (Figure 4J and 4L), which is consistent with the previously reported data that active Notch1 signaling is required for the differentiation of astrocytes [2]. This suggests that the absence of Prox1 expression in astrocytes may be required for Notch1 receptor expression and astrocyte differentiation. This observation could also explain the difference in slope between the curves for *Prox1* and *Notch1* mRNA levels upon withdrawal of GFs (solid lines in Figure 4E and 4F, respectively), since under these conditions a significant number of astrocytes are generated that express *Notch1* but not *Prox1* and contribute to mRNA levels. Finally, in agreement with these in vitro differentiation assays, a similar expression pattern for Prox1 was observed in vivo in embryonic mouse spinal cord (Figure S5).

**Figure 5:**
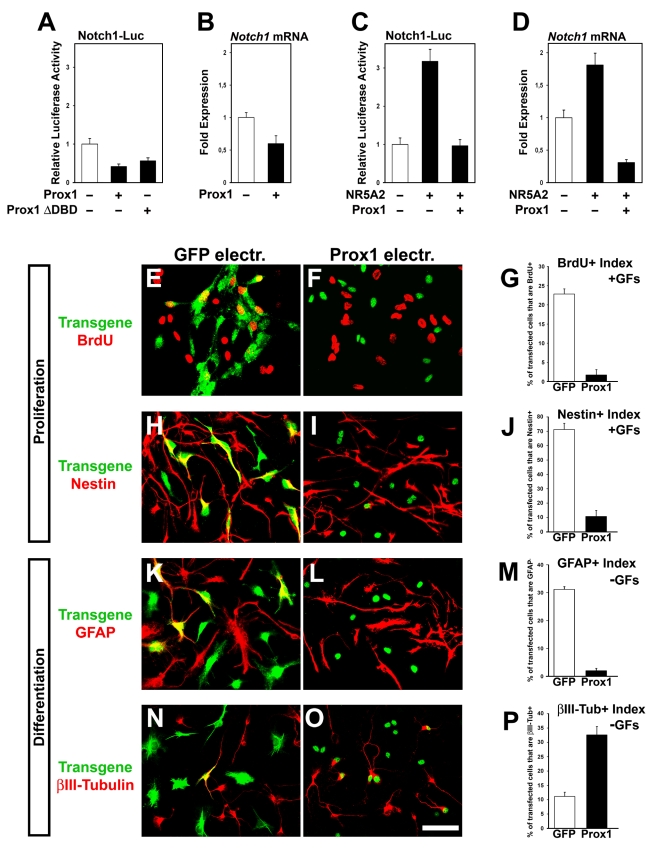
Forced expression of Prox1 in mouse NPCs suppresses progenitor identity, inhibits astrocyte differentiation, and induces neurogenesis. (A–D) Relative luciferase activities (A and C) and mRNA levels (B and D) were measured in NPCs transfected with various constructs as indicated. (E–G) Double GFP or Flag/BrdU immunostainings of NPCs in the presence of GFs and electroporated with GFP (E) or Prox1 (F) expression vectors. In all panels of this figure, expression of transgenes was detected with anti-GFP or anti-Flag antibodies, respectively. Quantification of BrdU index is shown in (G). *p*<0.001. (H–J) Double GFP or Flag/Nestin immunostainings of NPCs cultured in the presence of GFs and electroporated with GFP (H) or Prox1 (I) expression vectors. Quantification of Nestin index is shown in (J) *p*<0.001. (K–M) Double GFP or Flag/GFAP immunostainings of NPCs cultured in the absence of GFs and electroporated with GFP (K) or Prox1 (L) expression vectors. Quantification of GFAP index is shown in (M) *p*<0.001. (N–P) Double GFP or Flag/βIII-tubulin immunostainings of NPCs cultured in the absence of GFs and electroporated with GFP (N) or Prox1 (O) expression vectors. Quantification of βIII-tubulin index is shown in (P) *p*<0.01. For all panels scale bar: 50 µm.

To further test Prox1 function, we transfected NPCs with various constructs using the AMAXA electroporation system (Figure S6) [39]. We first verified that Prox1 suppresses *Notch1* transcription in these cells (Figure 5A and 5B). Similar to N2A data, the NR5A2 was sufficient to induce *Notch1* mRNA levels and Notch1-luc activity, while Prox1 was able to inhibit this induction (Figure 5C and 5D). To assess whether the inverse correlation between *Prox1* and *Notch1* expression has functional importance, we used NPCs as a model system to analyze the effect of Prox1 misexpression on Notch1-mediated self-renewal/proliferation and/or differentiation of NPCs (Figure 4M) [2],[40]. We first examined the effect of *Prox1* overexpression on NPC identity and proliferation under conditions that favor self-renewal (+GFs) (Figure S6). BrdU incorporation analysis 48 h after plating, followed by 2 h of BrdU-pulse, revealed a strong reduction in BrdU incorporation by 92.5% in a cell-autonomous manner (Figure 5E–5G). Similarly, a dramatic decrease by 85% in the proportion of Nestin+ cells was specifically observed in Prox1 transfected cells (Figure 5H–5J). Therefore, both indices show that Prox1 negatively affects self-renewal and proliferation of NPCs, with no indication of increased cell death as estimated by staining for activated caspase 3 (unpublished data). We next asked whether Prox1 overexpression also influences astrogliogenesis and neurogenesis. Under differentiation conditions, generation of astrocytes was severely impaired (by ∼90%) in the Prox1-electroporated NPCs, as evidenced by measuring the index of GFAP+ cells (Figure 5K–5M). Conversely, a significant increase in the proportion of βIII-tubulin+ cells by ∼3-fold was observed in the Prox1 electroporated cells (Figure 5N–5P). Collectively our results demonstrate that Prox1 through its action to negatively regulate the expression of *Notch1* is sufficient in a cell-autonomous manner to arrest self-renewal of Nestin+ NPCs and enhance their differentiation towards the acquisition of early βIII-tubulin+ neuronal identity, as opposed to depletion of GFAP+ astrocytes.

### Ectopic Expression of *Prox1* Suppresses *Notch1* In Vivo to Regulate Self-Renewal and Differentiation of NPCs

**Figure 6:**
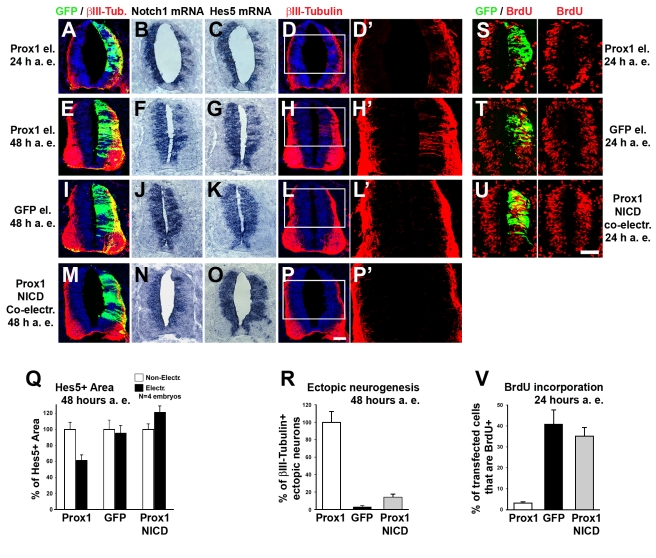
Misexpression of *Prox1* in vivo suppresses *Notch1* gene expression to induce early neuronal differentiation. (A–P) Double GFP/βIII-tubulin immunostainings (A, D, E, H, I, L, M, and P) and in situ hybridizations for *Notch1* (B, F, J, and N) and *Hes5* (C, G, K, and O) in consecutive sections, 24 h a. e. (A–D) or 48 h a.e. (E–P) with Prox1/GFP (A–H), GFP alone (I–L), or co-electroporation with Prox1/GFP and NICD (M–P). (D'), (H'), (L'), and (P') micrographs are larger magnifications of the white rectangle in (D), (H), (L), and (P), respectively. Scale bar: 50 µm. (Q) Quantitative analysis of the Hes5+ area presented in (G), (K), and (O) using the ImageJ software. The data are presented as % of non-electroporated side. For Prox1 versus GFP alone, *p*<0.01. For Prox1+NICD versus GFP alone, *p*<0.05. For Prox1+NICD versus Prox1, *p*<0.01. All cases referred to the electroporated side. (R) Percentage of ectopic βIII-tubulin+ neurons per embryo (10 sections per embryo; *n* = 4 embryos). The data are presented as % of Prox1 electroporated embryos. *p*<0.001 for Prox1 versus GFP; *p*<0.001 for Prox1 versus Prox1+NICD. (S–U) Double GFP/BrdU immunostainings 24 h a.e. with Prox1/GFP (S), GFP alone (T), or co-electroporation with Prox1/GFP and NICD (U), followed by 2-h BrdU pulse. Scale bar: 50 µm. (V) Quantitative analysis of BrdU incorporation. The number of BrdU+ transfected cells 24 h a.e. is expressed as percentage of the total number of transfected cells (*n* = 5 embryos; *p*<0.001 for Prox1 versus GFP; *p*<0.001 for Prox1 versus Prox1+NICD).

To further evaluate this conclusion in vivo, we misexpressed Prox1 unilaterally in the neural tube by in ovo electroporation of HH stage 12–14 (E2) chick embryos, following a previously reported protocol [39]. A striking reduction in the expression of *Notch1* at the mRNA level was observed in the neural tube of Prox1-electroporated embryos at both 24 h and 48 h after electroporation (a. e.), as compared to control GFP-transfected embryos (Figure 6A, 6B, 6E, 6F, 6I, and 6J). A concomitant reduction in expression of the *Notch1* target gene *Hes5* was also evident (Figure 6C, 6G, 6K, and 6Q), suggesting that Notch signaling is counteracted by Prox1 in a cell-autonomous manner. In addition, no evidence of apoptosis was observed either at 24 h or later at 48 h a.e. (Figure S7A and unpublished data), excluding the possibility that cells were depleted due to an apoptotic effect of Prox1. To further test whether the Prox1-mediated effect on *Notch1* expression is responsible for *Hes5* down-regulation, we co-expressed together with Prox1 the constitutively active intracellular domain of mammalian Notch1 (NICD) (Figure S7B–S7D). Co-expression of NICD was sufficient to rescue the negative effect of Prox1 on *Hes5* expression, excluding a direct action of Prox1 on *Hes5* transcription or another pathway (Figure 6M–6O and 6Q). We have previously shown that forced depletion of *Notch1* gene expression in the VZ results in ectopic induction of neurogenesis [39],[41]. Similarly, misexpression of Prox1 caused ectopic neuronal differentiation, as evidenced by induction of βIII-tubulin, an early marker of post-mitotic neurons (Figure 6D, 6H, 6L, and 6R). This observation has been previously reported by another group [20]. However, here, we wanted to examine whether Prox1 achieves its effect on neuronal differentiation via *Notch1* gene suppression. To this end, we again co-expressed NICD together with Prox1. Interestingly, NICD strongly inhibited the Prox1-mediated generation of ectopic βIII-tubulin+ neurons in the VZ (Figure 6P and 6R).

We then asked whether the capacity of Prox1 to suppress *Notch1* might affect self-renewal and proliferation of NPCs in the VZ. As previously reported [20], a striking reduction in the number of BrdU-incorporating cycling progenitors was observed in the neural tube of Prox1-transfected embryos 24 h a.e. compared with control GFP-transfected embryos (Figure 6S, 6T, and 6V). Similar to neuron differentiation, we showed here that co-expression of NICD abolishes the ability of Prox1 to arrest self-renewal of NPCs (Figure 6U–6V), indicating that Prox1-mediated suppression of Notch1 is essential for this function. In agreement, it was previously shown that Prox1 misexpression in chick spinal cord reduced the numbers of cycling Pax6+ and Pax7+ NPCs in the VZ [20]. Taken together our results support the hypothesis that Prox1 is involved in the transition of NPCs from self-renewal to neuronal differentiation via direct regulation of *Notch1*.

**Figure 7:**
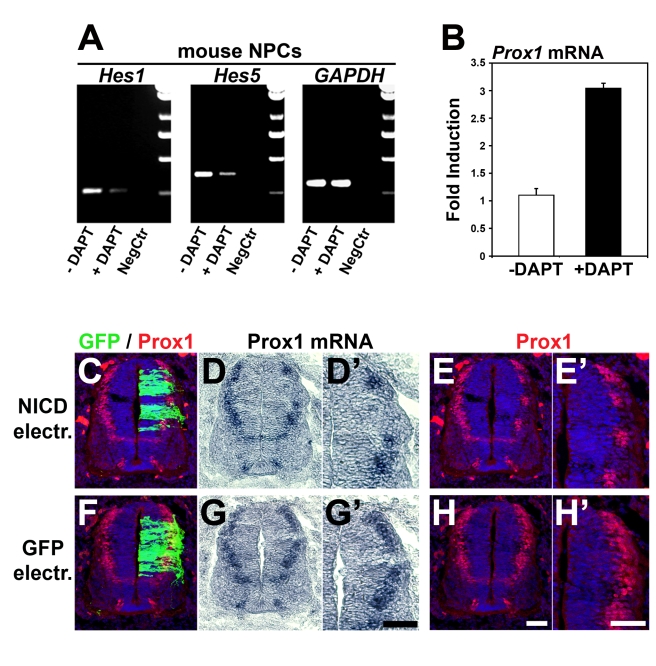
Active Notch1 signaling suppresses *Prox1* gene expression. (A–B) RT-PCR analysis of NPCs cultured in the presence or absence of DAPT, for *Hes1*, *Hes5*, *Gapdh* (A), and *Prox1* (B) genes, as indicated. For *Prox1* mRNA, *p*<0.01, *n* = 3. (C–H') Double GFP/Prox1 immunostainings and in situ hybridizations for *Prox1* in consecutive sections, as indicated, 48 h a.e. with NICD/GFP (C–E') or GFP alone (F–H'). (D'), (E'), (G'), and (H') micrographs are larger magnifications of the electroporated area in (D), (E), (G), and (H), respectively. Scale bar: 50 µm.

Given the opposite functions of Prox1 and Notch1 in NPCs, we then directly addressed the possibility that Notch1 antagonizes *Prox1* expression in a cross-inhibitory manner. To examine this, we first used the γ-secretase inhibitor DAPT in mouse NPCs. We verified that this treatment inactivated Notch signaling, as evidenced with reduced expression of *Hes1* and *Hes5* genes (Figure 7A). Under these conditions inhibition of Notch signaling caused a significant induction in endogenous *Prox1* mRNA expression by ∼3-fold (Figure 7B), suggesting that active Notch signaling in NPCs suppresses *Prox1* expression. Furthermore, when we misexpressed NICD in chick neural tube, we observed that ectopic activation of Notch1 signaling was sufficient to down-regulate endogenous expression of *Prox1* in vivo (Figure 7C–7H'). We conclude from these experiments that a cross-inhibitory interaction between Prox1 and active Notch1 signaling regulates the expression of both genes. The balance of this cross-inhibition might also regulate self-renewal and differentiation of NPCs.

### *Prox1* Expression Is Necessary for Suppression of *Notch1* In Vivo

**Figure 8:**
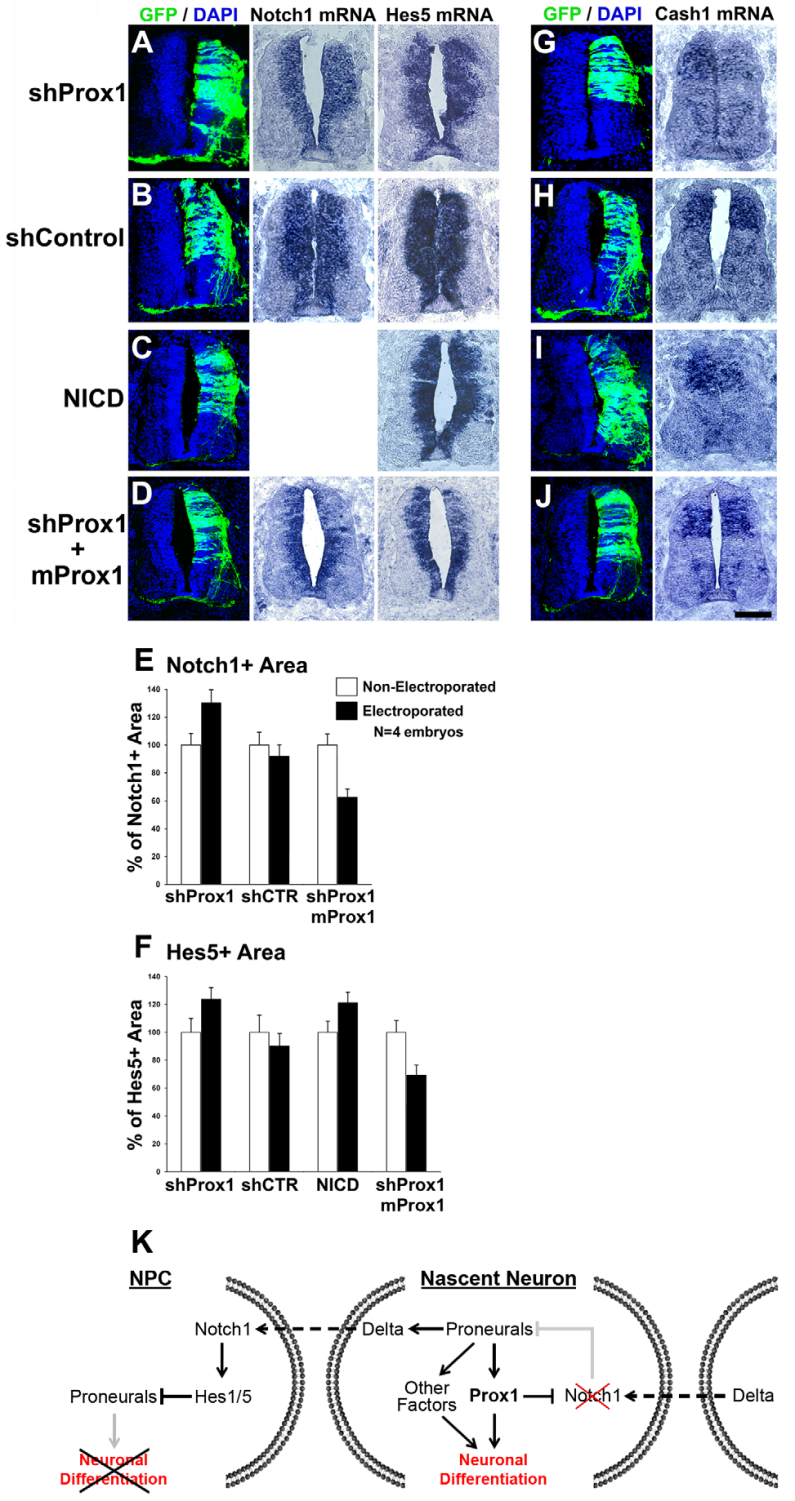
shRNA-mediated inhibition of *Prox1* expression in chick spinal cord enhances expression of *Notch1* and *Hes5* genes and impairs neurogenesis. (A–D) GFP/DAPI stainings and in situ hybridizations for *Notch1* and *Hes5* in consecutive sections 48 h a.e. with shProx1 (A), shControl (B), NICD+GFP (C), or shProx1+mProx1 (D). (E–F) Quantitative analysis of the Notch1+ (E) and Hes5+ (F) areas presented in (A–D) using the ImageJ software. The data are presented as % of non-electroporated side of the spinal cord. For Notch1+ area (E), shProx1 versus shControl, *p*<0.01; shProx1 versus shProx1+mProx1, *p*<0.01, *n* = 4 embryos. For Hes5+ area (F), shProx1 versus shControl, *p*<0.05; shProx1 versus NICD, *p*>0.1; shProx1 versus shProx1+mProx1, *p*<0.01, *n* = 4 embryos. All cases referred to the electroporated side. (G–J) GFP/DAPI stainings and in situ hybridization for *Cash1* in consecutive sections 48 h a.e. with shProx1 (G), shControl (H), NICD/GFP (I), or shProx1+mProx1 (J). Scale bar: 100 µm. (K) Schematic representation of the role of Prox1-mediated suppression of Notch1 expression in neuronal differentiation. During neurogenic phase of CNS development, NPCs divide asymmetrically to produce one NPC and one neuronal precursor/nascent neuron. In NPC, active Notch1 signaling prevents neuronal differentiation via direct inhibition of proneural genes. In nascent neurons, proneural genes activate *Prox1* to suppress *Notch1* gene expression and thus prevent activation of Notch1 receptor from neighboring signal-sending cells and sustain the program for neuronal differentiation. Moreover, Prox1-mediated inhibition of Notch1 may also block the inhibitory action of active Notch1 signaling on the expression of proneural genes, thus generating a positive feedback loop to maintain proneural gene expression and further enhance neuronal differentiation.

We next examined the requirement of Prox1 for suppressing *Notch1* and thus regulating neurogenesis in vivo. To inhibit *Prox1* expression in the chick neural tube, we utilized a pCAGGs-based shRNA system fused with GFP reporter gene to follow the expression of shRNAs (Figure S8A) [42],[43]. The effective knock-down of endogenous chick *Prox1* in the neural tube was assessed in mRNA and protein levels, by in situ hybridization and antibody staining, respectively (Figure S8B, S8C, S8F, and S8G). In addition, inhibition of endogenous *Prox1* did not affect programmed cell death, as evidenced with TUNEL assay (Figure S8J). Most important, knock-down of *Prox1* clearly induced the expression domain of *Notch1* towards the MZ by 41.8%, as compared to shControl (Figure 8A, 8B, and 8E). This induction was accompanied by a concomitant induction in *Hes5* gene expression in a manner similar to the induction caused by constitutively active NICD misexpression, indicative of ectopic Notch signaling activation (Figure 8A–8C and 8F). Consistently, expression of the proneural gene *Cash1* was reduced in response to *Prox1* depletion in a similar manner to NICD overexpression, indicative of enhanced Notch signaling that suppresses the initial phases of neurogenesis (Figure 8G–8I).

To exclude off-target effects and assess the specificity of Prox1-shRNA phenotypes, we performed two types of control experiments. First, we electroporated a control shRNA construct, containing a nucleotide sequence that does not target chick Prox1 and has no effect on chick Prox1 expression (Figure S8D, S8E, S8H, and S8I). This control construct did not alter the expression of *Notch1*, *Hes5* (Figure 8B, 8E, and 8F), or *Cash1* (Figure 8H). Second, the Prox1-shRNA construct was co-electroporated with a rescue construct containing the murine Prox1 coding sequence, which is quite divergent from the chick sequence and therefore is not targeted by the Prox1-shRNA sequence. A 6xHIS epitope tag was also included to be able to follow its expression (Figure S9A–S9C). Co-expression of murine Prox1 was sufficient to block the Prox1-shRNA-mediated induction of *Notch1* and *Hes5*, as well as the suppression of *Cash1* (Figure 8D, 8E, 8F, and 8J). Instead, it was able to revert the *Notch1* and *Hes5* phenotypes by suppressing the expression of both genes. Collectively, these lines of evidence strongly support that depletion of endogenous Prox1 is responsible for the effects observed on the expression of *Notch1*, *Hes5*, and *Cash1*.

Furthermore, we addressed whether shRNA-mediated knock-down of Prox1 affects the expression of other markers for NPCs. Thus, we examined the expression of the homeodomain factors, Pax7, Pax6, and Nkx2.2, which subdivide the VZ in the dorso-ventral axis, into defined progenitor domains with restricted developmental potentials. In each domain, whether dorsal or ventral, an induction in the number of cells that express these markers was observed in areas where Prox1-shRNA construct was misexpressed (Figure S10). Conversely, it was previously shown that forced Prox1 expression in chick spinal cord reduced the number of Pax7+ and Pax6+ NPCs [20]. Interestingly, Prox1 knock-down expanded the expression of Pax7, Pax6, and Nkx2.2 in the medio-lateral axis, towards the MZ, without affecting their dorso-lateral boundaries of expression. This suggests that Prox1 affects fundamental properties of NPCs relating to proliferation versus differentiation decisions, without interfering with their sub-type identity. In all cases overexpression of NICD phenocopied the effect of Prox1 knock-down (Figure S10E–S10G, S10L–S10N, and S10S–S10U), further suggesting that Prox1 is required for suppression of NPC markers in spinal cord via inhibition of Notch signaling.

These data indicate that Prox1-shRNA suppresses the initial phases of neurogenesis. To evaluate this conclusion, we examined expression of early and late markers of post-mitotic neurons, namely βIII-tubulin and SCG10, respectively. Our analysis was focused on dorsal interneurons, since Prox1 is mainly expressed in interneuron precursors (Figure 1 and Figure S1) [20]. As expected, Prox1 depletion strongly impaired neuronal differentiation by ∼50% (Figure S11A, S11E, S11F, and S11J), while no effect was seen in control shRNA transfected embryos (Figure S11B, S11E, S11G, and S11J). As previously reported [44], we also showed that NICD overexpression impaired neuronal differentiation, and thus it is sufficient to recapitulate the effect of Prox1 ablation on interneuron differentiation in spinal cord (Figure S11C, S11E, S11H, and S11J). Moreover, co-expression of murine Prox1 with shProx1 was able to rescue the negative effect on neurogenesis, since the numbers of βIII-tubulin+ and SCG10+ cells were restored (Figure S11D, S11E, S11I, and S11J). Taken together these results suggest that Prox1 is necessary for proper regulation of *Notch1* gene expression, through which it controls induction of interneuron differentiation.

## Discussion

In contrast with the wealth of information on the role of Notch signaling in neural development [1]–[3],[45], little is known of the upstream molecular mechanisms that control *Notch1* gene expression and regulate its inactivation in NPCs during neuronal differentiation [10],[46]. We show here that Prox1 directly interacts with the chromatin over the *Notch1* promoter in vivo, and physiological levels of endogenous Prox1 are critical for the proper regulation of *Notch1* gene expression in vivo. In particular, this study shows that Prox1 exerts a repressive action on *Notch1* mRNA expression in the boundaries between VZ and MZ during the neurogenic phase of vertebrate spinal cord development. This repressive action is sufficient for proper regulation of cell cycle exit and initiation of neuronal differentiation in NPCs, as evidenced by induction of βIII-tubulin, an early marker for post-mitotic neurons. In addition, Prox1 activity is necessary for suppression of active Notch signaling, down-regulation of NPC markers, and completion of neuronal differentiation program. We propose that Prox1 is directly involved in the molecular mechanism that couples and coordinates termination of Notch1 signaling with induction of neuronal differentiation (Figure 8K). It was previously shown that Notch1 signaling is modulated by a number of other mechanisms such as glycosylation, differential trafficking, and ubiquitin-dependent degradation of receptors and ligands, all of which play important roles in CNS development [2],[3],[47],[48]. However, as early neuronal precursors begin to differentiate and migrate outside the VZ, they cease to express *Notch1* at the mRNA level [11]–[13]. Thus, mechanisms that inhibit Notch1 receptor activity via protein modifications and/or stability in neuronal precursors may function as an early and immediate response in the VZ, whereas gene inactivation could act later in a more permanent way when cells migrate out of the VZ. Consistently, Numb, which acts as a Notch1 inhibitor via protein-protein interactions, begins to be expressed in neuronal precursors while in the VZ [49]–[51], whereas Prox1 is expressed later in the neuronal lineage when cells withdraw from the cell cycle and exit the VZ [20],[22],[25],[40]. Moreover, recent observations indicate that NPCs in the undifferentiated/self-renewing state express Notch ligands, such as Dll1, in an oscillatory manner [1], indicating that Notch ligands are constantly available in the neuroepithelium. Thus, we suggest that Prox1, by suppressing *Notch1* gene expression, prevents newly produced neuronal precursors from receiving signals from Notch ligands present in the membrane of neighboring cells and allows them to complete and sustain a neuronal differentiation program (Figure 8K).

Consistently, *Prox1* is induced by proneural genes and is required for implementation of their neurogenic program [20],[22]. In particular, *Prox1* is co-expressed with *Mash1* and *Ngn2* in the subventricular zone of murine brain and chick spinal cord during the initial stages of neurogenesis [20],[22]. Overexpression of these factors in the chick spinal cord [20] or murine NPCs is sufficient to induce *Prox1* expression [22]. Conversely, *Prox1* levels are reduced in the embryonic brain of *Mash1* knockout mice [22], further suggesting that Prox1 expression during neurogenesis is dependent on proneural genes. This epistatic relationship between proneural genes and *Prox1* may also explain the inhibitory action of active Notch signaling on *Prox1* expression (Figure 7), since active Notch signaling directly suppresses the expression of proneural genes [6],[44], which then cannot induce *Prox1* (Figure 8K). In support, we were able to identify a number of conserved E-box sequences on the *Prox1* gene locus representing putative proneural protein binding sites (Figure S12). However, based on our data we cannot exclude the possibility of a direct action of Notch signaling on *Prox1* expression.

Moreover, this epistatic relationship could also explain how proneural genes, after a certain number of oscillatory cycles of expression, manage to overcome in a cell-autonomous manner the Notch1-mediated inhibition on neurogenesis without depleting NPCs [1]. In this regard, an intriguing question is why oscillations of proneural genes, such as Ngn2, in NPCs are unable to induce neuronal differentiation whereas their sustained expression does elicit differentiation. It has been proposed that only a subset of downstream genes, perhaps those already expressed in the VZ, respond quickly to changes in proneural gene expression, whereas genes expressed outside the VZ respond more slowly and cannot be induced when Ngn2 expression oscillates [1]. Thus, Prox1 may belong to these slowly responsive genes, induced upon sustained Ngn2 expression to terminate Notch1 signaling and facilitate neuronal differentiation. In this scenario, NPCs are not depleted despite short pulses of Ngn2 expression but are maintained by sustaining activated Notch signaling, which in turn prevents neuronal differentiation.

Although Prox1 acts downstream of the bHLH proneural genes [20] and is able to promote early neurogenic events, such as ectopic inactivation of Notch signaling, cell cycle exit of NPCs, and ectopic induction of βIII-tubulin+ cells in the VZ, it is not sufficient to induce a full neurogenic expression program [20]. This observation suggests that Prox1 acts in concert with other factors downstream of proneural proteins to induce neurogenesis (Figure 8K). In agreement, proneural proteins are capable to promote the expression of multiple downstream factors, such as NeuroD, NeuroM, Nscl1, Delta1, Cend1, and Sox4/11, which are involved in the implementation of a full neurogenic cascade [5],[39],[52],[53]. These factors are sufficient to induce a partial array of neuronal specific phenotypes that can be fully achieved by bHLH proneural factors. For example, Sox4/11 promote the expression of specific neuronal markers, but they are not sufficient to induce the exit of NPCs from the cell cycle or suppress progenitor-specific gene expression [52]. Conversely, Prox1 is able to achieve the latter but cannot fully phenocopy the effect of Sox4/11 on inducing neuronal markers. Thus, proneural proteins appear to activate multiple complementary downstream programs to potentiate full neuronal differentiation. Moreover, genetic or pharmacological inhibition of Notch signaling in chick neural tube is sufficient to induce proneural gene expression, cause NPCs to exit the cell cycle, downregulate progenitor identities, and induce a full neurogenic expression program [44]. Although Prox1 misexpression in the same system is able to inhibit Notch signaling, it cannot precisely mimic the effect of Notch inhibition on terminal neuronal differentiation [20]. These observations suggest that Prox1 exerts an extra action on NPCs, which is not compatible with terminal neuronal differentiation in the spinal cord. Consistently, endogenous *Prox1* expression is downregulated prior to acquisition of terminal neuronal identity in this region of the CNS [20]. In addition to this function, Prox1 might be involved in the specification of neuronal sub-types, since its expression is excluded from the pMN domain (MN progenitors) of the ventral spinal cord. In agreement, genetic inactivation of *Notch1* from NPCs of the ventral spinal cord suppresses MN identity and induces V2 interneurons, suggesting that Notch1 activity is required for generation of MNs [54]. Thus, Prox1 exclusion from pMN could be associated with this requirement. Furthermore, Prox1 is also expressed in several other regions of the CNS, apart from the spinal cord, during embryonic and postnatal stages of development, including the cortex, dentate gyrus, thalamus, and cerebellum [21],[55],[56]. Interestingly, in adult brain, Prox1 expression remains high in mature neurons of the hippocampus and cerebellum [55]. This expression pattern is quite distinct from the transient pattern of Prox1 expression in the embryonic spinal cord, suggesting that Prox1 function may be different in the developing or mature nervous system [55]–[57]. Given that Prox1 is a pleiotropic factor affecting many diverse signaling pathways and transcriptional networks in other tissues and organs [16],[24],[26],[27],[29],[34],[35],[36], this differential expression pattern may also imply a different mechanism of Prox1 action in these regions of CNS. Therefore, based on our observations in the embryonic spinal cord, we cannot rule out the possibility that other signaling pathways may be involved in mediating the function of Prox1 in other CNS regions independently of its ability to counteract Notch1 in a cell-autonomous manner. In agreement, it was recently published that ablation of *Prox1* in postnatal dentate gyrus leads to an increase in apoptosis of intermediate progenitors and the absence of adult neurogenesis through a non-cell autonomous mechanism [57].

A key question arising from our observations is whether other Notch receptors (Notch2, 3, and 4) are implicated in the Prox1-mediated inactivation of Notch signaling as documented by *Hes5* gene down-regulation. Although, based on our data, we cannot exclude a possible function of Prox1 in inhibiting other Notch receptors, the fact that overexpression of the constitutively active intracellular domain of Notch1 is sufficient to overcome the effect of Prox1 on *Hes5* gene down-regulation, cell-cycle exit, and ectopic neuronal differentiation of NPCs suggests that these effects may be achieved primarily through the Notch1 receptor. Moreover, previous studies that report the phenotypes of *Notch1*, *2*, *3*, and *4* gene deletions in mouse embryos imply Notch1 as the most important Notch receptor in CNS development and differentiation [3]. *Notch3* and *Notch4* deletions do not affect neuronal differentiation or CNS development [58]–[60]. On the other hand, *Notch2* ^−/−^ embryos die around E11 and undergo massive cell death in the CNS similar to *Notch1* ^−/−^ mice. However, they do not show alterations in *Hes5* expression in striking contrast to *Notch1* ^−/−^ mutants [61],[62]. Consistently, *Notch1* and *Notch2* are differentially expressed in mouse embryonic CNS [12],[63].

Moreover, transcriptional assays in cell lines and NPCs indicate that Prox1 acts as a transcriptional co-repressor in suppressing *Notch1* and its action may be mediated by NR5A2 and HDAC3 proteins that are co-expressed and interact with Prox1. We suggest that these proteins facilitate Prox1 recruitment on Notch1 promoter and chromatin-mediated transcriptional repression, respectively. Despite the fact that both proteins are expressed in primary NPCs and embryonic mouse and chick CNS, their role in CNS development is not known. Interestingly, the Prox1/NR5A2 complex, where Prox1 acts as a co-repressor, plays a crucial role in the proper regulation of a set of genes, which are very important for liver development, regeneration, and function [27],[35],[36],[64],[65], suggesting that this role might have been conserved in CNS development and function. In support of this hypothesis, we showed here that physiological levels of endogenous Prox1 and NR5A2 are sufficient to allow binding at the *Notch1* promoter locus in the embryonic mouse CNS, and most important, re-ChIP assays in the same tissue suggest that Prox1 and NR5A2 could form a complex on chromatin over the *Notch1* promoter.

To conclude, in this study we have unveiled for the first time to our knowledge a novel means of regulation of Notch signaling in the developing spinal cord, which involves transcriptional repression of *Notch1* by Prox1. In addition, we have demonstrated that this transcriptional repression has profound implications in cell cycle exit and differentiation of neuronal precursors as they exit the VZ and migrate towards the MZ to acquire terminally differentiated phenotypes. This mechanism is of paramount importance for generating the correct number of neurons from a duly sustained pool of NPCs and we would like to propose that part of the important roles of Prox1 in many different aspects of embryonic development, organ morphogenesis, and cancer pathogenesis [23],[25]–[29],[66] may be mediated through its ability to counteract Notch signaling.

## Materials and Methods

### Ethics Statement

All animals were handled in strict accordance with good animal practice as defined by the relevant European and Greek animal welfare bodies.

### RNA Extraction and Real-Time RT-PCR Analysis

Total RNA was isolated by using the RNAeasy Kit (Qiagen) followed by treatment with RQ1 DNase. Quantitative real time RT-PCR analysis was performed as described [39].

Primer sets used in RT-PCR assays:

mProx1-For: CAGCGGACTCTCTAGCACAG

mProx1-Rev: GCCTGCCAAAAGGGGAAAGA

mNotch1-For: GCCGCAAGAGGCTTGAGAT

mNotch1-Rev: GGAGTCCTGGCATCGTTGG

mHes1-For: TCAACACGACACCGGACAAACC

mHes1-Rev: GGTACTTCCCCAACACGCTCG

mHes5-For: CTCCGCTCCGCTCGCTAATCGC

mHes5-Rev: GCTTCATCTGCGTGTCGCTGGC

mNR5A2-For: TGAGTGGGCCAGGAGTAGTA

mNR5A2-Rev: ATCAAGAGCTCACTCCAGCA

mHDAC3-For: TATGCAGGGTTTCACCAAGA

mHDAC3-Rev: CAGAGATGCGCCTGTGTAAC

mGAPDH-For: AACTCCCTCAAGATTGTCAGCAA

mGAPDH-Rev: ATGTCAGATCCACAACGGATACA

### Luciferase Assays

Transient transfections and luciferace reporter assays were performed with Lipofectamine (Invitrogen) and luciferase/β-galactosidase kits (Promega), respectively, as previously described [67],[68]. For Notch1-luc, Hes1-luc, Hes5-luc, and TK-luc constructs we have used 0.4 µg per transfection and 1.6 µg of expression vectors (*Prox1*, *NR5A2*, or *HDAC3*). All experiments were done in quadruplicate at least three times, and statistical analysis was performed by the paired two-sample Student's *t* test.

### Targeted Mutagenesis of the Notch1-Luciferase Promoter Construct

To generate the mut-N1-Luc a PCR-based approach was used, with the following primer sets:

N1promoter-EXT-For: AAGTAAGCTTCTTGGGGGAGCGGGGCACA

N1promoter-EXT -Rev: TCTTCCATGGGCCTCCCCACCGGCT

N1promoter-INT-For: CCGCCCCGGGATAATACGATTATTCACATGCAAATTTCA

N1promoter-INT-Rev: ATGTGAATAATCGTATTATCCCGGGGCGGAATGGGGA

By this approach the two overlapping Prox1 consensus binding sites [33] on Notch1 promoter were mutated from CTCCTCCGCT to ATAATACGAT.

To generate the 535-N1-Luc construct, a BamHI/NcoI fragment from the WT Notch1-Luc construct was digested and inserted into the parental pGL-2basic vector. All plasmid constructs were verified with sequencing.

### ChIPs

ChIP assays were performed essentially as previously described with minor modifications [69]. 10 to 25 µg of chromatin were used per IP reaction with up to 3 µg of antibody. Chomatin-antibody immunocomplexes were formed using affinity purified antibodies to Prox1 (ReliaTech, 102-PA32), HDAC3 (Santa Cruz Biotechnology, SC-11417) [70], and NR5A2 (kindly provided by Dr Talianidis) [71]. Antibody bound chromatin was retained on protein A/G-magnetic beads (Invitrogen). DNA was extracted from the immobilized bound immunocomplexes reversed, ethanol precipitated, and analyzed by semi-quantitative PCR. The following primer pairs were used to amplify the promoter and ORF genomic loci from mouse *Notch1* gene as indicated in Figure 2D:

N1-Promoter-5: AGTGCCTGGCCTCAATCCTCC (21 bp)

N1-Promoter-3: TGTAGCCGCCCTCTGCGACAT (21 bp)

N1-ORF-5: GCCAGTACAACCCACTACGG (20 bp)

N1-ORF-3: CACTGAGGTGTGGCTGTGAT (20 bp)

The re-ChIP experiments were performed as previously reported [71]. Briefly, the immunocomplexes were pulled down with the first antibody, treated with 20 mM DTT, followed by a 20-fold dilution before performing the immunoprecipitation with the second antibody. Cross-linking was reversed and DNA was purified and subjected to PCR using the above mentioned primer sets.

### Co-Immunoprecipitation Assay

Co-immunoprecipitation assays were performed from embryonic mouse CNS tissue (E12.5), including both brain and spinal cord. Tissues were lysed in 20 mM Tris HCl (pH 7,5), 140 mM NaCl, 1% Νοnidet-P40, 2 mM EDTA, 1 mM Phenylmethylsulfonyl fluoride (PMSF), and cocktail inhibitors (SIGMA). The following antibodies were used: anti-HDAC3 (Santa Cruz Biotechnology, SC-11417) [70], anti-Brd2 (Santa Cruz Biotechnology, SC-46805), anti-Prox1 (Chemicon, Mab5654), and control IgGs from DAKO. To retain antibodies protein-A agarose beads were utilized (Pierce).

### In Situ Hybridization on Cryosections

Non-radioactive in situ hybridization on cryosections and preparations for digoxigenin- or fluorescein-labeled probes were carried out as described [39],[72].

### Immunohistochemistry

Prox1 was detected using a rabbit polyclonal anti-Prox1 antibody (ReliaTech) or a mouse monoclonal antibody (Chemicon). Anti-BrdU monoclonal antibody was purchased from Dako and detected as previously described [39]. Anti-Nestin and anti-O4 monoclonal antibodies were from Chemicon; monoclonal anti-GFAP was from Sigma; anti-activated caspase 3, anti-HDAC3, anti-Brd2, and anti-Notch1 from Santa Cruz; and monoclonal anti-βIII-tubulin was from Covance (USA). Anti-GFP and anti-FLAG were purchased from Molecular Probes and Sigma, respectively. Pax6, Pax7, and Nkx2.2 antibodies were obtained from Developmental Studies Hybridoma Bank (University of Iowa, Iowa City). Detection of cells undergoing apoptosis on sections was carried out with the TUNEL kit from Roche (Mannheim, Germany) using Streptavidin Texas Red (Amersham). Secondary antibodies conjugated with AlexaFluor 488 (green) or 546 (red) were from Molecular Probes. Cell nuclei were labeled with Hoechst 33258 or DAPI (1∶1000, Molecular Probes). Specimens were viewed and analyzed with a Leica confocal microscope. Statistical analysis was performed with the two-tailed paired Student's *t* test.

### Culture of NPCs and Overexpression Studies

NPCs cultures from embryonic mouse spinal cords were performed as previously described [39], with some modifications. NPCs were prepared from E14.5 mouse embryo spinal cords and maintained as neurosphere cultures in a serum-free medium [1∶1 mixture of DMEM and F-12 with penicillin (100 units/ml; Invitrogen) and streptomycin (100 µg/ml; Invitrogen)], containing the B-27 supplement (1 ml/50 ml medium; Invitrogen), insulin (20 µg/ml; Sigma), recombinant human basic fibroblast growth factor (bFGF 20 ng/ml; R&D Systems), and epidermal growth factor (EGF 20 ng/ml; R&D Systems ). After 5–7 d in culture, floating neurospheres were trypsin-dissociated and allowed to re-form spheres at least three times before further use.

Proliferation studies were performed after dissociation to single cells, plating onto poly-l-lysine coated coverslips in 24-well plates at a density of 6×10^4^, and further culture for 2 d in the presence of EGF/bFGF. For differentiation, dissociated neurospheres were plated on poly-l-lysine-coated coverslips at a density of 7×10^4^and maintained for 2 or 3 d in the absence of growth factors.

For Prox1 or GFP overexpression, cells were transfected using an AMAXA electroporator (Lonza). The pCDNA3-Prox1 and control GFP mammalian expression vectors were used, driving transgene expression under the control of CMV promoter (5 µg of plasmid DNA per electroporation). Transfected neurospheres were cultured for 24 h, after which, either they were enzymatically dissociated and plated as single cells in poly-l-lysine-coated 10-mm-diameter coverslips or they were cultured for a further 24 h and collected for RNA extraction, luciferase assays, or Real-Time RT-PCR analysis.

For quantification of proliferation, the index of BrdU+ cells was determined by scoring the transgene (Prox1 or GFP) and BrdU double-positive cells versus the total number of transgene positive cells for each set of electroporations (Prox1 and GFP) from five independent experiments. For quantification of differentiation, the indices of Nestin+, βIII-tubulin+, GFAP+, and O4+ cells were determined by scoring the transgene (Prox1 or GFP) and Nestin or βIII-tubulin or GFAP or O4 double-positive cells versus the total number of transgene positive cells for each set of electroporations (Prox1 and GFP) from five independent experiments. Statistical analysis was performed by the paired two-sample Student's *t* test.

### In Ovo Electroporation

Unilateral overexpression of transgenes and shRNA constructs in the chick neural tube by in ovo electroporation method was performed as previously described [39]. Briefly, white Leghorn chicken eggs were incubated at 38 °C until stages 12–14 of development (E2). Supercoiled plasmid for electroporation was used at a concentration of 1–2 mg/ml in TE (10 mM Tris-HCl, 1 mM EDTA, pH 7.5) with 0.025% Fast Green (Sigma). Embryos were injected with DNA solution into the lumen of the neural tube and then subjected to electroporation. The electrodes were spaced 4 mm apart and positioned such that the DNA was driven into the cells on only one side of the neural tube. Embryos were pulsed 2×3 times for 30 ms each at 28 V. Eggs were then re-incubated for 1 or 2 d before the embryos were fixed for 4 h at 4 °C in 4% paraformaldehyde in PBS. Embryos were then washed in PBS, cryoprotected with 20% sucrose, mounted in OCT (Tissue-Tek), and sectioned at 12–14 µm. For BrdU-labeling, embryos received 10 µg BrdU in PBS 2 h before fixation. To distinguish the expression of exogenous transgenes from that of the endogenous genes, pCaggs based expression constructs were co-electroporated alongside a GFP expression plasmid. To create pCAGGs-Prox1 and pCaggs-NICD expression vectors, the respective mouse and human cDNAs were cloned into pCAGGS empty vector.

For the construction of the shRNA vectors targeting chick *Prox1*, we followed previously published methods [42],[43]. In particular, to generate the appropriate vectors we used the following primer sets exactly as previously described [42]:

W: GGCGGGGCTAGCTGGAGAAGATGCCTTCCGGAGAGGTGCTGCTGAGCG

Y: GGGTGGCTTAAGAAGAGGGGAAGAAAGCTTCTAACCCCGCTATTCACCACCACTAGGCA

Target: CTTCCTGGAAGAAGGCCATATA

RNAi-cProx1-B-For: GAGAGGTGCTGCTGAGCGATTCCTGGAAGAAGGCCATATATAGTGAAGCCACAGATGTA

RNAi-cProx1-B-Rev: ATTCACCACCACTAGGCACTTCCTGGAAGAAGGCCATATATACATCTGTGGCTTCACT

Target sequences on *cProx1* were identified with a genescript free online tool as described [42].

## Supporting Information

Figure S1 **Comparison of spatiotemporal expression patterns of** ***Prox1*** **,** ***Notch1*** **,** ***Hes5*** **, and** ***SCG10*** **in early embryonic chick and mouse spinal cord.** (A–P) Adjacent transverse sections of HH stage 12 (A–D), HH stage 18 (E–H), HH stage 24 (I–L), and HH stage 29 (M–P) cervical spinal cords were hybridized with *Prox1*, *Notch1*, *Hes5*, and *SCG10* riboprobes (specific for chick), respectively, as indicated. *Prox1* mRNA expression is initiated after HH stage 12 (A) and remained until HH stage 29 (E, I, and M). Note the inverse correlation between *Prox1* expression and *Notch1*, as well as *Hes5* expression, in all stages examined. Scale bars: 40 µm (A–L); 100 µm (M–P). (Q–T) Horizontal section of mouse spinal cord were cut as indicated in the schematic drawing in the left and were co-stained with anti-Prox1 (red) and anti-Notch1 (green) antibodies in combination with DAPI staining to reveal cell nuclei. Confocal analysis revealed that the majority of cells do not co-express Prox1 and Notch1. However, there are few cases that Prox1 and Notch1 are expressed in the same cells (white arrows in T). The midline is indicated. Scale bar: 50 µm.
(8.46 MB TIF)

Figure S2 **Prox1-mediated transcriptional repression of** ***Notch1*** **gene promoter in N2A cells.** (A) Transcriptional assays in N2A cells co-transfected with Notch1-Luc construct and human *Prox1* expression construct or empty vector. Data are represented as the mean ± SD of quadruplicate assays (*p*<0.01). (B) In the upper panel: Western blot analysis of WT Prox1 or ΔDBD Prox1 overexpression in N2A cells. Both proteins are tagged with the Flag epitope. Protein detection was performed with an anti-Flag antibody and anti-Actin for loading control. In the lower panel: anti-Flag (green) immunostainings of N2A cells transfected with WT Prox1 or ΔDBD Prox1. Scale bar: 25 µm. (C) In the upper panel: Western blot analysis of ΔRD Prox1, 331 Prox1, or 131 Prox1 overexpression in N2A cells, as indicated. All proteins are tagged with the Flag epitope. Protein detection was performed with an anti-Flag antibody. In the lower panel: anti-Flag immunostainings of N2A cells transfected with the indicated vectors. Scale bar: 25 µm. (D) Transcriptional assays in N2A cells co-transfected with Notch1-Luc construct and either *NR5A2* or *SF1* expression vectors, as indicated. Data are represented as the mean ± SD of quadruplicate assays. For WT versus NR5A2, *p*<0.01; WT versus SF1, *p*<0.05. (E) Transcriptional assays in N2A cells co-transfected with Notch1-Luc construct and *NR5A2* in the presence of either WT Prox1 or ΔRD Prox1. Data are represented as the mean ± SD of quadruplicate assays. For WT versus NR5A2, *p*<0.01; WT versus NR5A2/Prox1, *p*>0.1; WT versus NR5A2/ΔRD-Prox1, *p*<0.01; NR5A2/Prox1 versus NR5A2/ΔRD-Prox1, *p*<0.01. (F) Transcriptional assays in N2A cells transfected with Notch1-Luc and treated with either 150 nM TSA or vehicle alone, as indicated. Data are represented as the mean ± SD of quadruplicate assays (*p*<0.001). (G) Transcriptional assays in N2A cells co-transfected with Notch1-Luc and WT Prox1 or ΔDBD Prox1 and treated with either 150 nM TSA or vehicle alone, as indicated. Data are represented as the mean ± SD of quadruplicate assays. For WT versus Prox1/TSA, *p*<0.01; Prox1 versus Prox1/TSA, *p*<0.01; Prox1 versus ΔDBD-Prox1/TSA, *p*<0.01.
(3.37 MB TIF)

Figure S3 **Expression pattern of NR5A2 in embryonic mouse spinal cord.** (A–H) Transverse sections of E12.5 (A–D), E14.5 (E–F), and E16.5 (G–H) embryonic mouse spinal cords were co-stained with anti-NR5A2 and anti-βIII-tubulin (B, F, and H) or anti-Nestin (D) antibodies, as indicated. (B'), (D'), (F'), and (H') micrographs are larger magnifications of the white rectangle in (B), (D), (F), and (H), respectively. Arrows in (B'), (D'), (F'), and (H') indicate NR5A2+ cells that co-express βIII-tubulin (B', F', and H') or Nestin (D'). Note that NR5A2 expression is detected in βIII-tubulin+ neurons of the mantle zone (A–B', arrows in B') and Nestin+ NPCs of the ventricular zone (C–D', arrows in D'). Scale Bars: 100 µM (B, D, F, and H); 50 µM (B', D', F', and H').
(9.12 MB TIF)

Figure S4 **Expression pattern of HDAC3 in embryonic mouse spinal cord.** (A) RT-PCR analysis in NPCs cultured in vitro, and E12.5 mouse spinal cords, for the detection of *Hdac3* and *Gapdh* mRNAs, as indicated. (B–E') Transverse sections of E10.5 (B–D') and E12.5 (E–E') embryonic mouse spinal cords were co-stained with anti-HDAC3 and anti-Prox1 antibodies, as indicated. (D') and (E') micrographs are larger magnifications of the white rectangle in (D) and (E), respectively. Note that the majority of Prox1+ cells express HDAC3. Arrows in (D') and (E') indicate Prox1+ cells that co-express HDAC3. NPCs, neural progenitor cells; SC, Spinal Cord; M, marker. Scale bar: 100 µM.
(9.48 MB TIF)

Figure S5 **Expression pattern of Prox1 in embryonic mouse spinal cord.** (A–G) Transverse sections of E10.5 (A–B), E12.5 (C–E), and E16.5 (F–G) embryonic mouse spinal cords were co-stained with anti-Prox1 and anti-βIII-tubulin (A, C, and F) or anti-Nestin (B and D) or anti-O4 (E) or anti-GFAP (G) antibodies, as indicated. (A'), (B'), (C'), (D'), (F'), and (G') micrographs are larger magnifications of the white rectangle in (A), (B), (C), (D), (F), and (G), respectively. Arrows in (A'), (B'), (C'), (D'), (E), and (F') indicate Prox1+ cells that co-express βIII-tubulin (A', C', and F'), Nestin (B' and D'), or O4 (E). Arrowheads in (A'), (B'), (D'), (E), and (G') indicate Prox1+ cells that are not positive for βIII-tubulin (A'), Nestin (B' and D'), O4 (E), and GFAP (G'). Note that Prox1 is excluded from the GFAP+ astrocytes in E16.5 spinal cord (G–G'). Scale Bars: 100 µM (A, B, C, D, F, and G); 50 µM (A', B', C', D', F', and G').
(8.77 MB TIF)

Figure S6 **Schematic representation of the protocol used for AMAXA electroporation of NPCs.** Neurosphere cultures were passaged by enzymatic dissociation at least three times before electroporation. After electroporation NPCs were cultured for 24 h and then dissociated and plated in the presence or absence of GFs for 48 h to immunostain them for various markers and measure proliferation and differentiation indices. For measuring mRNA or luciferase activity, cells were lysed 48 h after electroporation and mRNA or protein extracts were prepared, respectively.
(1.66 MB TIF)

Figure S7 **Quantification of Apoptosis in Prox1/GFP, GFP alone, or Prox1/NICD co-electroporated embryos. (**A) Quantification of apoptosis in the electroporated and non-electroporated sides of the spinal cord 24 h a.e. with Prox1/GFP, GFP alone, or Prox1/GFP+NICD. Results are expressed as the numbers of TUNEL+ cells per embryo in the transfected area and compared with the TUNEL+ cells in the equivalent area of the non-transfected side (eight sections per embryo; *n* = 4 embryos; for all three cases, *p*>0.1). (B) Schematic drawing for the Prox1 and NICD expression constructs. (C–D) Double immunofluorescence analysis of a transverse section of chick embryo spinal cord 48 h a.e. with Prox1 and NICD, as indicated. Scale bar: 50 µm.
(3.73 MB TIF)

Figure S8 **Identification of a shRNA construct efficient in down-regulating endogenous** ***Prox1*** **expression in chick embryonic spinal cord.** (A) Schematic representation of the shRNA based constructs used in this study. GFP under the control of chick β-actin promoter was also included to follow expression of the shRNA. (B–E) GFP/DAPI stainings and in situ hybridization for *cProx1* gene in consecutive sections 48 h a.e. with shProx1 (B–C) or shControl (D–E). (F–I) Double GFP/Prox1 immunostainings 48 h a.e. with shProx1 (F–G) or shControl (H–I). Scale bar: 100 µm. (J) Quantification of apoptosis in the electroporated and non-electroporated sides of the spinal cord 48 h a.e. with shProx1 or shControl. Results are expressed as the numbers of TUNEL+ cells per embryo in the transfected area and compared with the TUNEL+ cells in the equivalent area of the non-transfected side (eight sections per embryo; *n* = 4 embryos; for both cases, *p*>0.1).
(9.03 MB TIF)

Figure S9 **Rescue of the shRNA-Prox1 mediated phenotypes by murine Prox1.** (A) Schematic representation of the shProx1 and murine Prox1 expression vectors. Note that shProx1 can be detected with GFP and murine Prox1 is tagged with 6xHIS epitope, and thus can be detected with anti-HIS immunostaining. (B–C) Double GFP/HIS immunostaining 48 h after co-electroporation of shProx1 and murine Prox1 constructs. Note that murine Prox1 is distributed in the same cells as the shProx1 construct. Scale bar: 100 µm.
(4.11 MB TIF)

Figure S10 **Prox1 is necessary for the suppression of Pax7, Pax6, and Nkx2.2 expression in the chick embryonic spinal cord.** (A–F) Double GFP/Pax7 immunostainings 48 h a.e. with shProx1 (A–B), shControl (C–D), or NICD/GFP (E–F). The white lines indicate the outline of the spinal cord. (B'), (D'), and (F') micrographs are larger magnifications of the electroporated area in (B), (D), and (F), respectively. Scale bar: 75 µm. (G) Quantitative analysis of the number of transfected cells (GFP+) that are Pax7+ (white columns) or Pax7- (black columns). The data are presented as % of the total number of transfected cells (GFP+), *n* = 4 embryos. For GFP+/Pax7−, shProx1 versus shControl, *p*<0.01; shControl versus NICD/GFP, *p*<0.01; shProx1 versus NICD/GFP, *p*>0.1. (H–M) Double GFP/Pax6 immunostainings 48 h a.e. with shProx1 (H–I), shControl (J–K), or NICD/GFP (L–L'). The white lines indicate the outline of the spinal cord. (I'), (K'), and (M') micrographs are larger magnifications of the electroporated area in (I), (K), and (M), respectively. Scale bar: 75 µm. (N) Quantitative analysis of the number of transfected cells (GFP+) that are Pax6+ (white columns) or Pax6− (black columns). The data are presented as % of the total number of transfected cells (GFP+), *n* = 4 embryos. For GFP+/Pax6−, shProx1 versus shControl, *p*<0.01; shControl versus NICD/GFP, *p*<0.01; shProx1 versus NICD/GFP, *p*>0.1. (O–T) Double GFP/Nkx2.2 immunostainings 48 h a.e. with shProx1 (O–P), shControl (Q–R), or NICD/GFP (S–T). Scale bar: 50 µm. (U) Quantitative analysis of the number of Nkx2.2+ cells presented in (O–T). The data are presented as % of non-electroporated side of the spinal cord. For shProx1 versus shControl, *p*<0.05; shControl versus NICD/GFP, *p*<0.05; shProx1 versus NICD/GFP, *p*>0.1, *n* = 4 embryos. All cases referred to the electroporated side. (V–W) Endogenous Prox1 is expressed in a subset of Nkx2.2+ cells in the ventral spinal cord. Double Prox1/Nkx2.2 immunostainings in transverse sections of wild type embryonic chick spinal cord of HH stage 24. Scale bar: 50 µm.
(9.97 MB TIF)

Figure S11 **shRNA-mediated inhibition of** ***Prox1*** **expression in chick spinal cord impairs neurogenesis.** (A–D) Double GFP/βIII-tubulin immunostainings 48 h a.e. with shProx1 (A), shControl (B), NICD+GFP (C), or shProx1+mProx1 (D). Scale bar: 100 µm. (E) Quantitative analysis of the βIII-tubulin+ areas presented in (A–D) using the ImageJ software. The data are presented as % of non-electroporated side of the spinal cord. shProx1 versus shControl, *p*<0.01; shControl versus NICD, *p*<0.01; shProx1 versus NICD, *p*>0.1; shProx1 versus shProx1+mProx1, *p*<0.01, *n* = 4 embryos. All cases referred to the electroporated side. (F–I) GFP/DAPI stainings and in situ hybridization for *SCG10* in consecutive sections 48 h a.e. with shProx1 (F), shControl (G), NICD+GFP (H), or shProx1+mProx1 (I). Scale bar: 100 µm. (J) Quantitative analysis of the SCG10+ area presented in (F–I) using the ImageJ software. The data are presented as % of non-electroporated side of the spinal cord. shProx1 versus shControl, *p*<0.01; shControl versus NICD, *p*<0.01; shProx1 versus NICD, *p*>0.1; shProx1 versus shProx1+mProx1, *p*<0.01, *n* = 4 embryos. All cases referred to the electroporated side.
(8.59 MB TIF)

Figure S12 **Schematic representation of the conserved binding sites (E-boxes) for proneural proteins on the mouse** ***Prox1*** **gene locus.** (A–B) 168 kb of the mouse *Prox1* gene locus (100 kb upstream and 20 kb downstream of the *Prox1* gene, chromosome 1: from 192094506 to 191926560) were aligned with the corresponding area of the human genome and the conserved binding sites for proneural proteins were identified as indicated (red lines). This analysis was performed with the ECR Browser software tool, freely available at http://ecrbrowser.dcode.org. Nine conserved putative binding sites were identified, based on the E-box consensus sequence, as previously published (CANNTG) [5],[53]. Detailed description of the orientation, position, and sequence of each site is indicated in (B).
(1.86 MB TIF)

## Abbreviations

a. e.: after electroporation
CNS: central nervous system
DBD: DNA binding domain
GF: growth factor
HDAC3: Histone Deacetylase 3
MZ: Mantle zone
NICD: Notch1 intracellular domain
NPCs: Neural progenitor cells
NR5A2: Nuclear Receptor 5A2
NSCs: Neural stem cells
MN: motor neuron
pMN: MN progenitor domain
RD: Repression Domain
SC: Spinal cord
SF1: Steroidogenic Factor 1
VZ: Ventricular zone
