# Supplementary figures and images for: Prox1 Regulates the Notch1-Mediated Inhibition of Neurogenesis
Source: PLoS Biol. 2010 Dec 21;8(12):e1000565. doi: 10.1371/journal.pbio.1000565 (PMC3006385; doi:10.1371/journal.pbio.1000565)

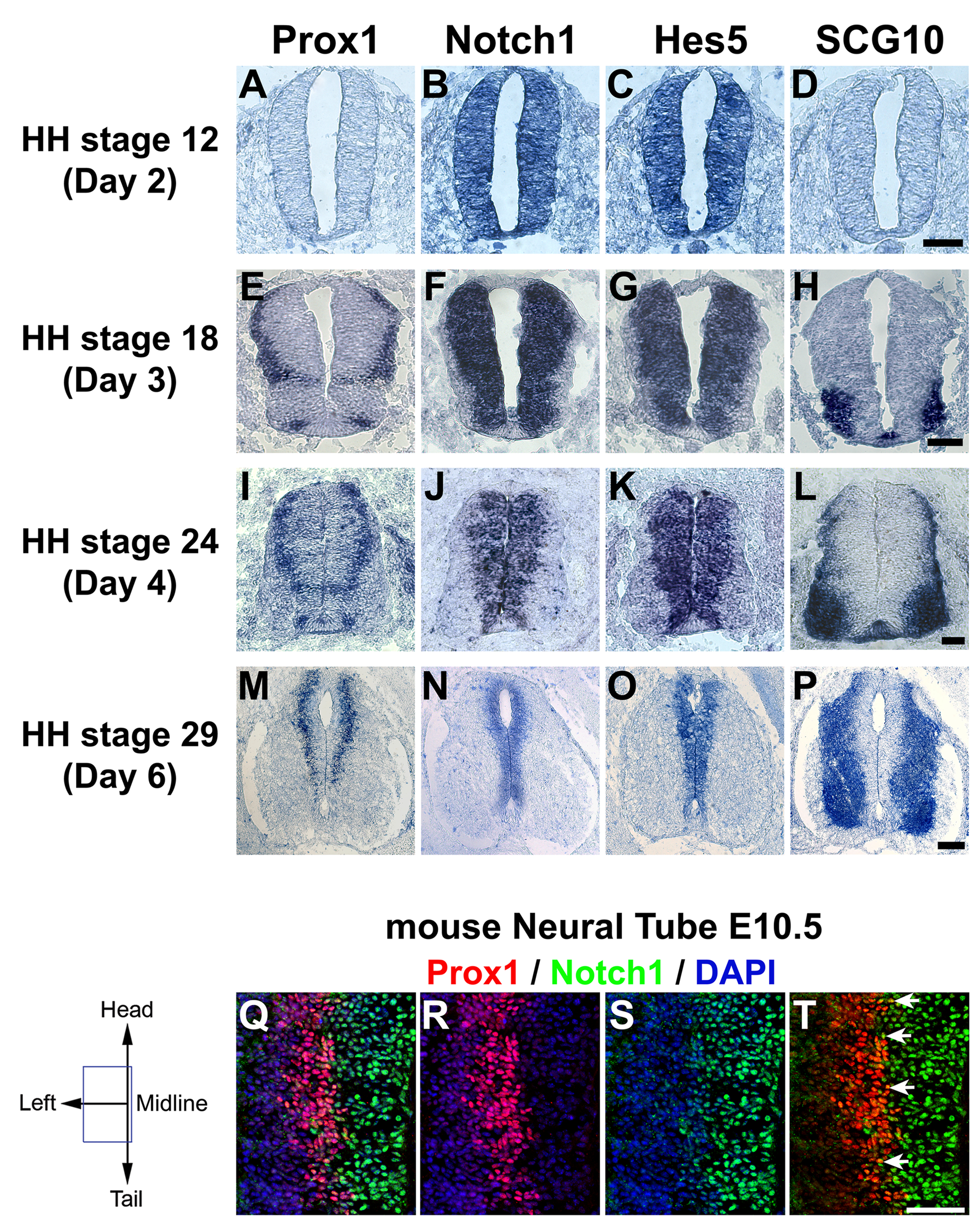

Supplement: Figure S1 — Comparison of spatiotemporal expression patterns of Prox1 , Notch1 , Hes5 , and SCG10 in early embryonic chick and mouse spinal cord. (A–P) Adjacent transverse sections of HH stage 12 (A–D), HH stage 18 (E–H), HH stage 24 (I–L), and HH stage 29 (M–P) cervical spinal cords were hybridized with Prox1, Notch1, Hes5, and SCG10 riboprobes (specific for chick), respectively, as indicated. Prox1 mRNA expression is initiated after HH stage 12 (A) and remained until HH stage 29 (E, I, and M). Note the inverse correlation between Prox1 expression and Notch1, as well as Hes5 expression, in all stages examined. Scale bars: 40 µm (A–L); 100 µm (M–P). (Q–T) Horizontal section of mouse spinal cord were cut as indicated in the schematic drawing in the left and were co-stained with anti-Prox1 (red) and anti-Notch1 (green) antibodies in combination with DAPI staining to reveal cell nuclei. Confocal analysis revealed that the majority of cells do not co-express Prox1 and Notch1. However, there are few cases that Prox1 and Notch1 are expressed in the same cells (white arrows in T). The midline is indicated. Scale bar: 50 µm. (8.46 MB TIF) [file pbio.1000565.s001.tif]

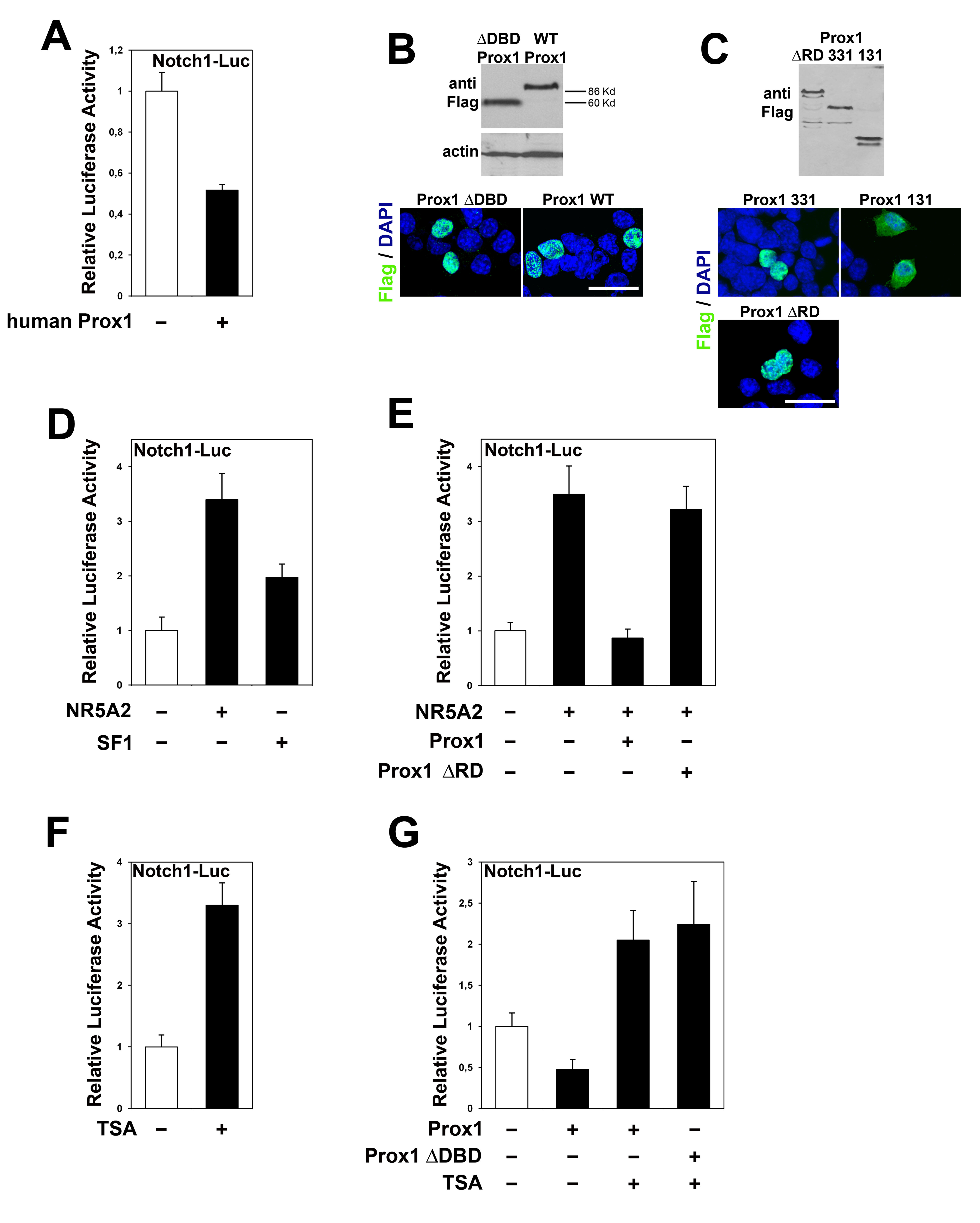

Supplement: Figure S2 — Prox1-mediated transcriptional repression of Notch1 gene promoter in N2A cells. (A) Transcriptional assays in N2A cells co-transfected with Notch1-Luc construct and human Prox1 expression construct or empty vector. Data are represented as the mean ± SD of quadruplicate assays (p<0.01). (B) In the upper panel: Western blot analysis of WT Prox1 or ΔDBD Prox1 overexpression in N2A cells. Both proteins are tagged with the Flag epitope. Protein detection was performed with an anti-Flag antibody and anti-Actin for loading control. In the lower panel: anti-Flag (green) immunostainings of N2A cells transfected with WT Prox1 or ΔDBD Prox1. Scale bar: 25 µm. (C) In the upper panel: Western blot analysis of ΔRD Prox1, 331 Prox1, or 131 Prox1 overexpression in N2A cells, as indicated. All proteins are tagged with the Flag epitope. Protein detection was performed with an anti-Flag antibody. In the lower panel: anti-Flag immunostainings of N2A cells transfected with the indicated vectors. Scale bar: 25 µm. (D) Transcriptional assays in N2A cells co-transfected with Notch1-Luc construct and either NR5A2 or SF1 expression vectors, as indicated. Data are represented as the mean ± SD of quadruplicate assays. For WT versus NR5A2, p<0.01; WT versus SF1, p<0.05. (E) Transcriptional assays in N2A cells co-transfected with Notch1-Luc construct and NR5A2 in the presence of either WT Prox1 or ΔRD Prox1. Data are represented as the mean ± SD of quadruplicate assays. For WT versus NR5A2, p<0.01; WT versus NR5A2/Prox1, p>0.1; WT versus NR5A2/ΔRD-Prox1, p<0.01; NR5A2/Prox1 versus NR5A2/ΔRD-Prox1, p<0.01. (F) Transcriptional assays in N2A cells transfected with Notch1-Luc and treated with either 150 nM TSA or vehicle alone, as indicated. Data are represented as the mean ± SD of quadruplicate assays (p<0.001). (G) Transcriptional assays in N2A cells co-transfected with Notch1-Luc and WT Prox1 or ΔDBD Prox1 and treated with either 150 nM TSA or vehicle alone, as indicated. Data are represented as [file pbio.1000565.s002.tif]

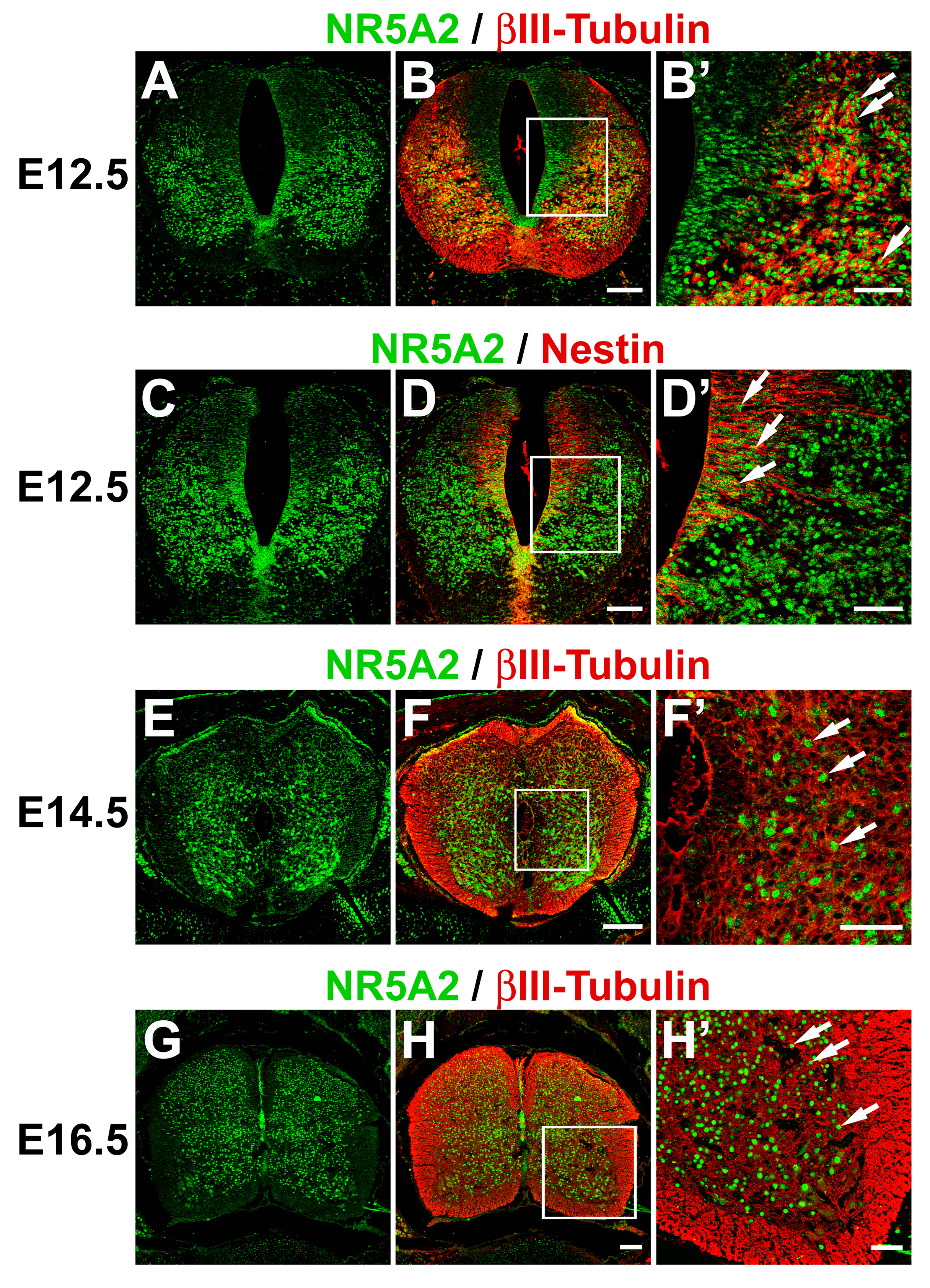

Supplement: Figure S3 — Expression pattern of NR5A2 in embryonic mouse spinal cord. (A–H) Transverse sections of E12.5 (A–D), E14.5 (E–F), and E16.5 (G–H) embryonic mouse spinal cords were co-stained with anti-NR5A2 and anti-βIII-tubulin (B, F, and H) or anti-Nestin (D) antibodies, as indicated. (B'), (D'), (F'), and (H') micrographs are larger magnifications of the white rectangle in (B), (D), (F), and (H), respectively. Arrows in (B'), (D'), (F'), and (H') indicate NR5A2+ cells that co-express βIII-tubulin (B', F', and H') or Nestin (D'). Note that NR5A2 expression is detected in βIII-tubulin+ neurons of the mantle zone (A–B', arrows in B') and Nestin+ NPCs of the ventricular zone (C–D', arrows in D'). Scale Bars: 100 µM (B, D, F, and H); 50 µM (B', D', F', and H'). (9.12 MB TIF) [file pbio.1000565.s003.tif]

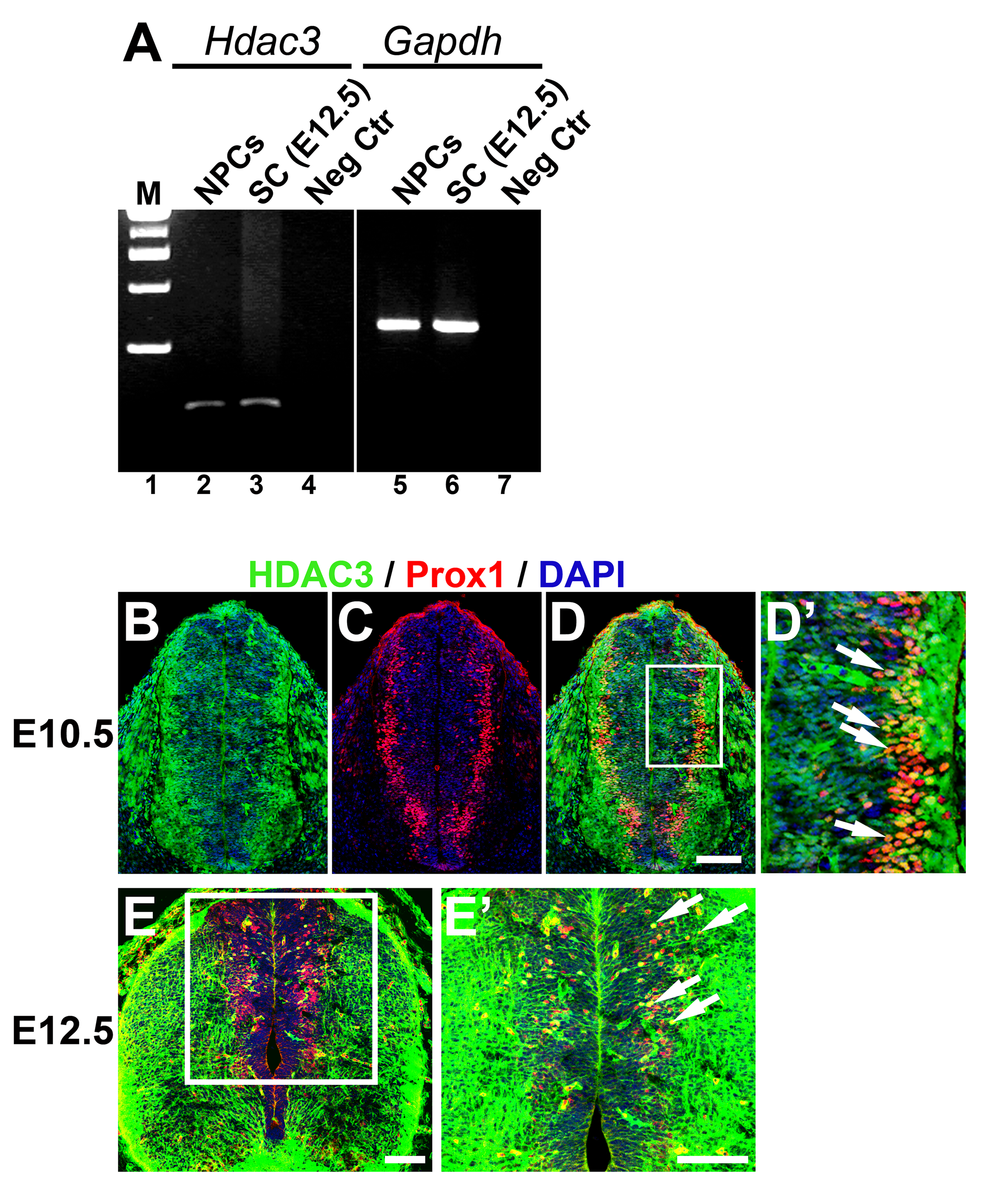

Supplement: Figure S4 — Expression pattern of HDAC3 in embryonic mouse spinal cord. (A) RT-PCR analysis in NPCs cultured in vitro, and E12.5 mouse spinal cords, for the detection of Hdac3 and Gapdh mRNAs, as indicated. (B–E') Transverse sections of E10.5 (B–D') and E12.5 (E–E') embryonic mouse spinal cords were co-stained with anti-HDAC3 and anti-Prox1 antibodies, as indicated. (D') and (E') micrographs are larger magnifications of the white rectangle in (D) and (E), respectively. Note that the majority of Prox1+ cells express HDAC3. Arrows in (D') and (E') indicate Prox1+ cells that co-express HDAC3. NPCs, neural progenitor cells; SC, Spinal Cord; M, marker. Scale bar: 100 µM. (9.48 MB TIF) [file pbio.1000565.s004.tif]

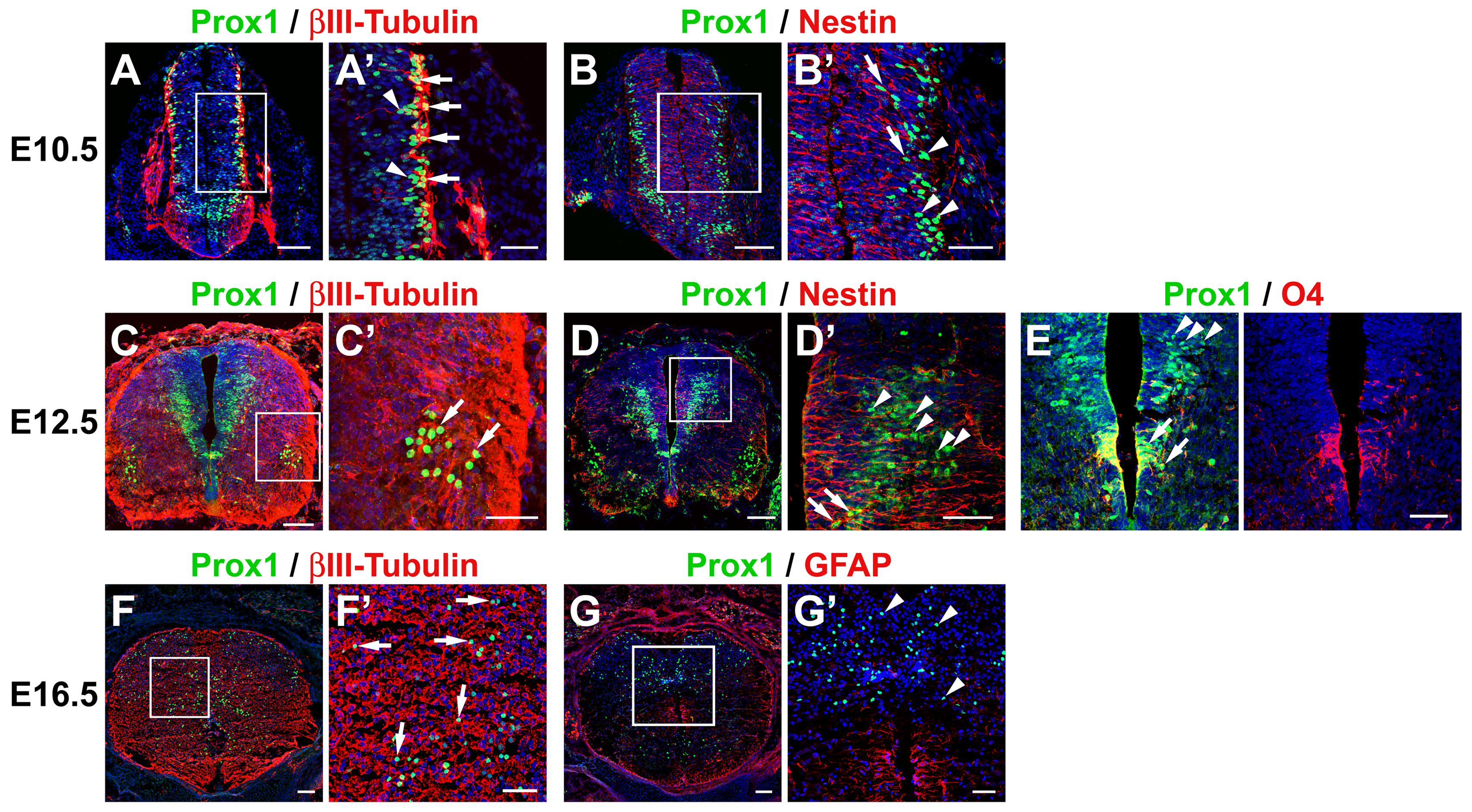

Supplement: Figure S5 — Expression pattern of Prox1 in embryonic mouse spinal cord. (A–G) Transverse sections of E10.5 (A–B), E12.5 (C–E), and E16.5 (F–G) embryonic mouse spinal cords were co-stained with anti-Prox1 and anti-βIII-tubulin (A, C, and F) or anti-Nestin (B and D) or anti-O4 (E) or anti-GFAP (G) antibodies, as indicated. (A'), (B'), (C'), (D'), (F'), and (G') micrographs are larger magnifications of the white rectangle in (A), (B), (C), (D), (F), and (G), respectively. Arrows in (A'), (B'), (C'), (D'), (E), and (F') indicate Prox1+ cells that co-express βIII-tubulin (A', C', and F'), Nestin (B' and D'), or O4 (E). Arrowheads in (A'), (B'), (D'), (E), and (G') indicate Prox1+ cells that are not positive for βIII-tubulin (A'), Nestin (B' and D'), O4 (E), and GFAP (G'). Note that Prox1 is excluded from the GFAP+ astrocytes in E16.5 spinal cord (G–G'). Scale Bars: 100 µM (A, B, C, D, F, and G); 50 µM (A', B', C', D', F', and G'). (8.77 MB TIF) [file pbio.1000565.s005.tif]

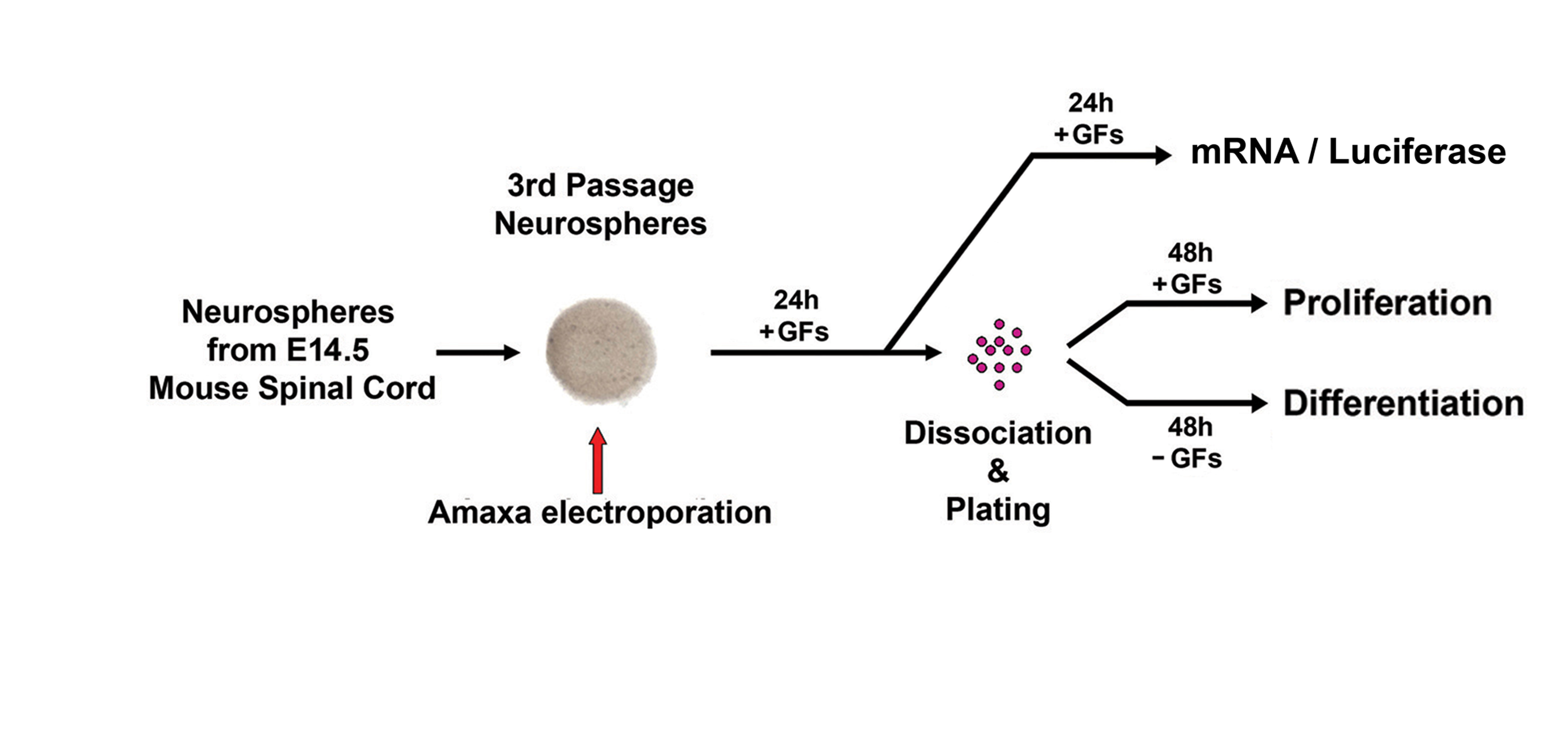

Supplement: Figure S6 — Schematic representation of the protocol used for AMAXA electroporation of NPCs. Neurosphere cultures were passaged by enzymatic dissociation at least three times before electroporation. After electroporation NPCs were cultured for 24 h and then dissociated and plated in the presence or absence of GFs for 48 h to immunostain them for various markers and measure proliferation and differentiation indices. For measuring mRNA or luciferase activity, cells were lysed 48 h after electroporation and mRNA or protein extracts were prepared, respectively. (1.66 MB TIF) [file pbio.1000565.s006.tif]

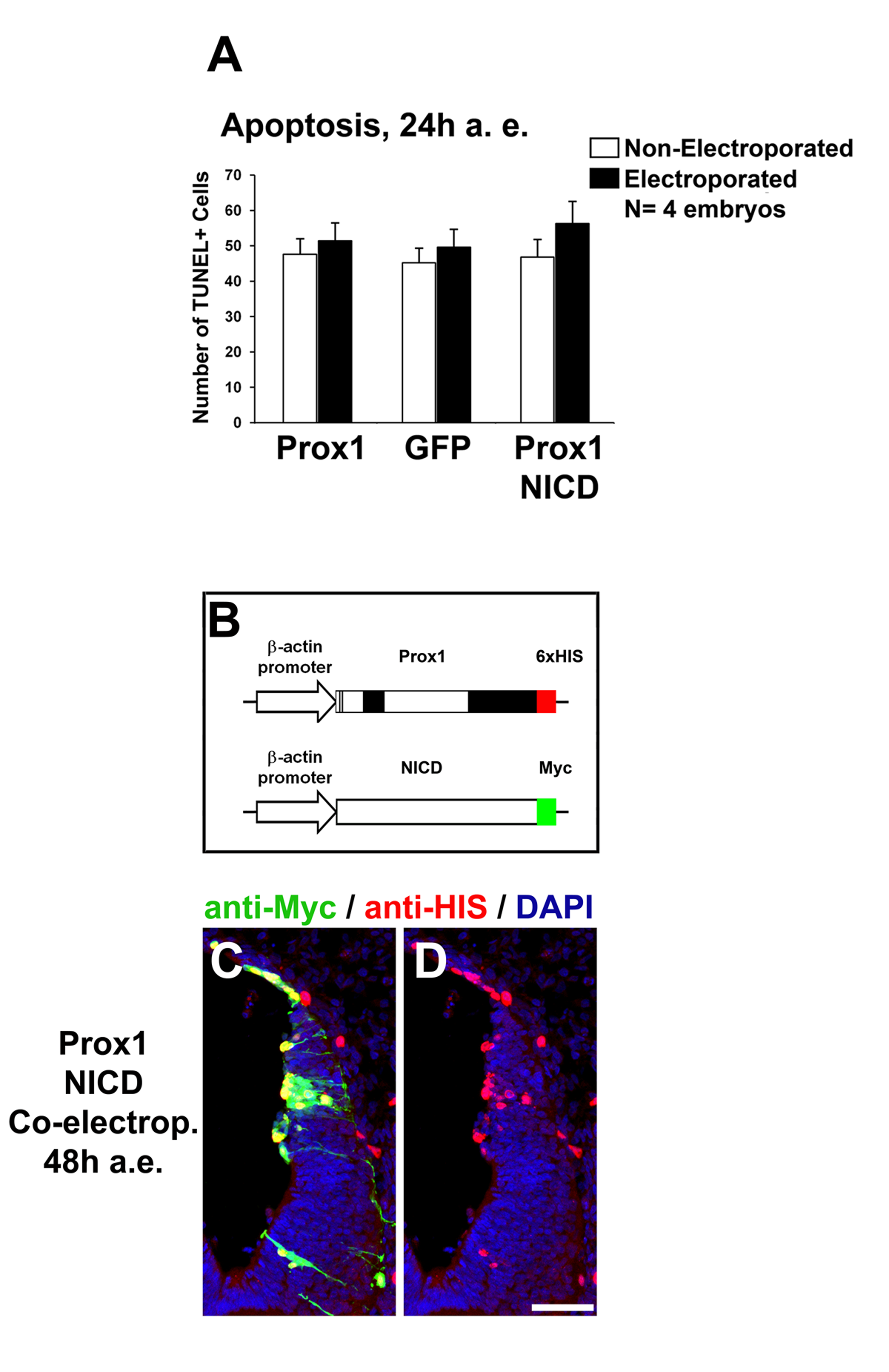

Supplement: Figure S7 — Quantification of Apoptosis in Prox1/GFP, GFP alone, or Prox1/NICD co-electroporated embryos. (A) Quantification of apoptosis in the electroporated and non-electroporated sides of the spinal cord 24 h a.e. with Prox1/GFP, GFP alone, or Prox1/GFP+NICD. Results are expressed as the numbers of TUNEL+ cells per embryo in the transfected area and compared with the TUNEL+ cells in the equivalent area of the non-transfected side (eight sections per embryo; n = 4 embryos; for all three cases, p>0.1). (B) Schematic drawing for the Prox1 and NICD expression constructs. (C–D) Double immunofluorescence analysis of a transverse section of chick embryo spinal cord 48 h a.e. with Prox1 and NICD, as indicated. Scale bar: 50 µm. (3.73 MB TIF) [file pbio.1000565.s007.tif]

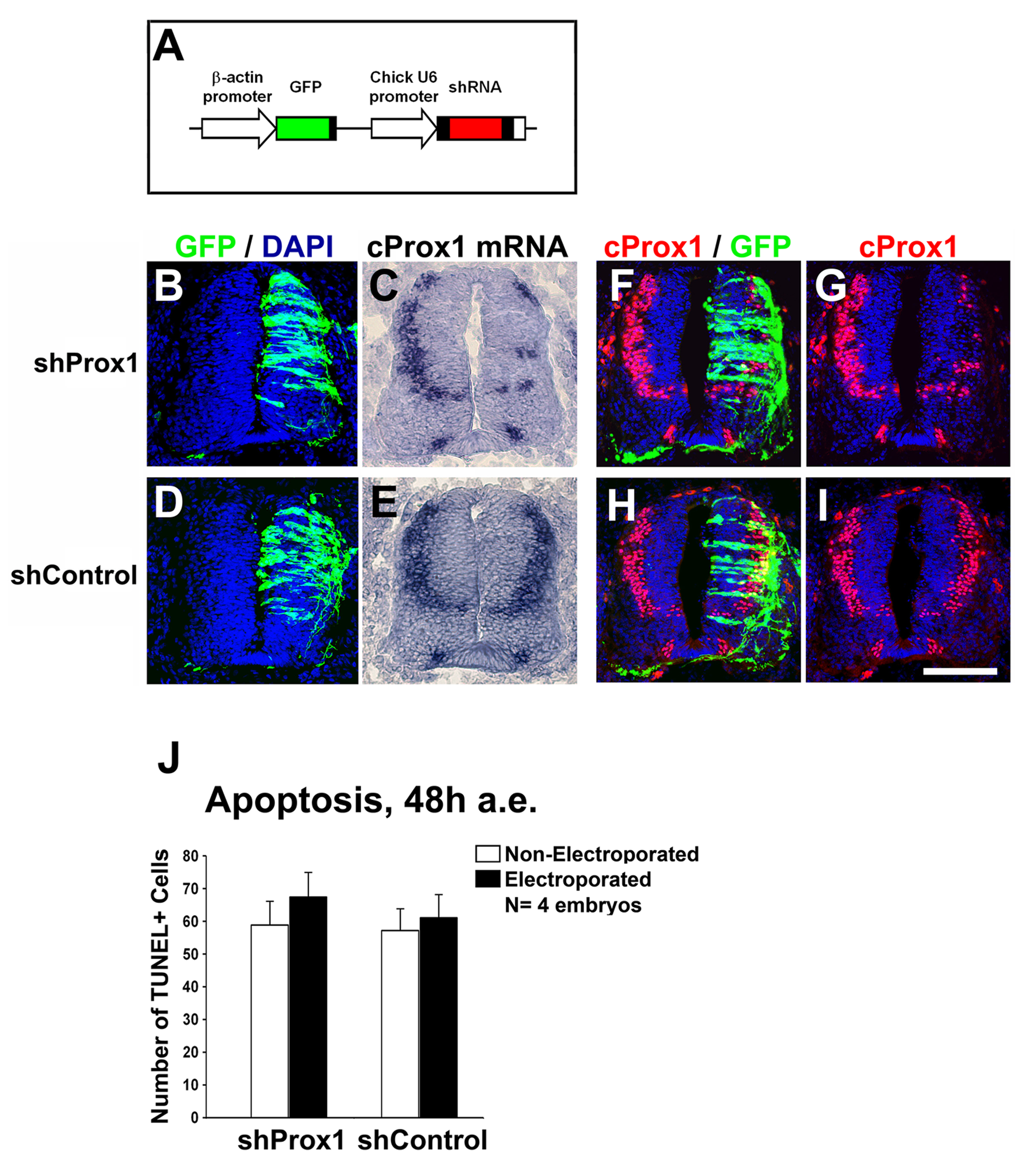

Supplement: Figure S8 — Identification of a shRNA construct efficient in down-regulating endogenous Prox1 expression in chick embryonic spinal cord. (A) Schematic representation of the shRNA based constructs used in this study. GFP under the control of chick β-actin promoter was also included to follow expression of the shRNA. (B–E) GFP/DAPI stainings and in situ hybridization for cProx1 gene in consecutive sections 48 h a.e. with shProx1 (B–C) or shControl (D–E). (F–I) Double GFP/Prox1 immunostainings 48 h a.e. with shProx1 (F–G) or shControl (H–I). Scale bar: 100 µm. (J) Quantification of apoptosis in the electroporated and non-electroporated sides of the spinal cord 48 h a.e. with shProx1 or shControl. Results are expressed as the numbers of TUNEL+ cells per embryo in the transfected area and compared with the TUNEL+ cells in the equivalent area of the non-transfected side (eight sections per embryo; n = 4 embryos; for both cases, p>0.1). (9.03 MB TIF) [file pbio.1000565.s008.tif]

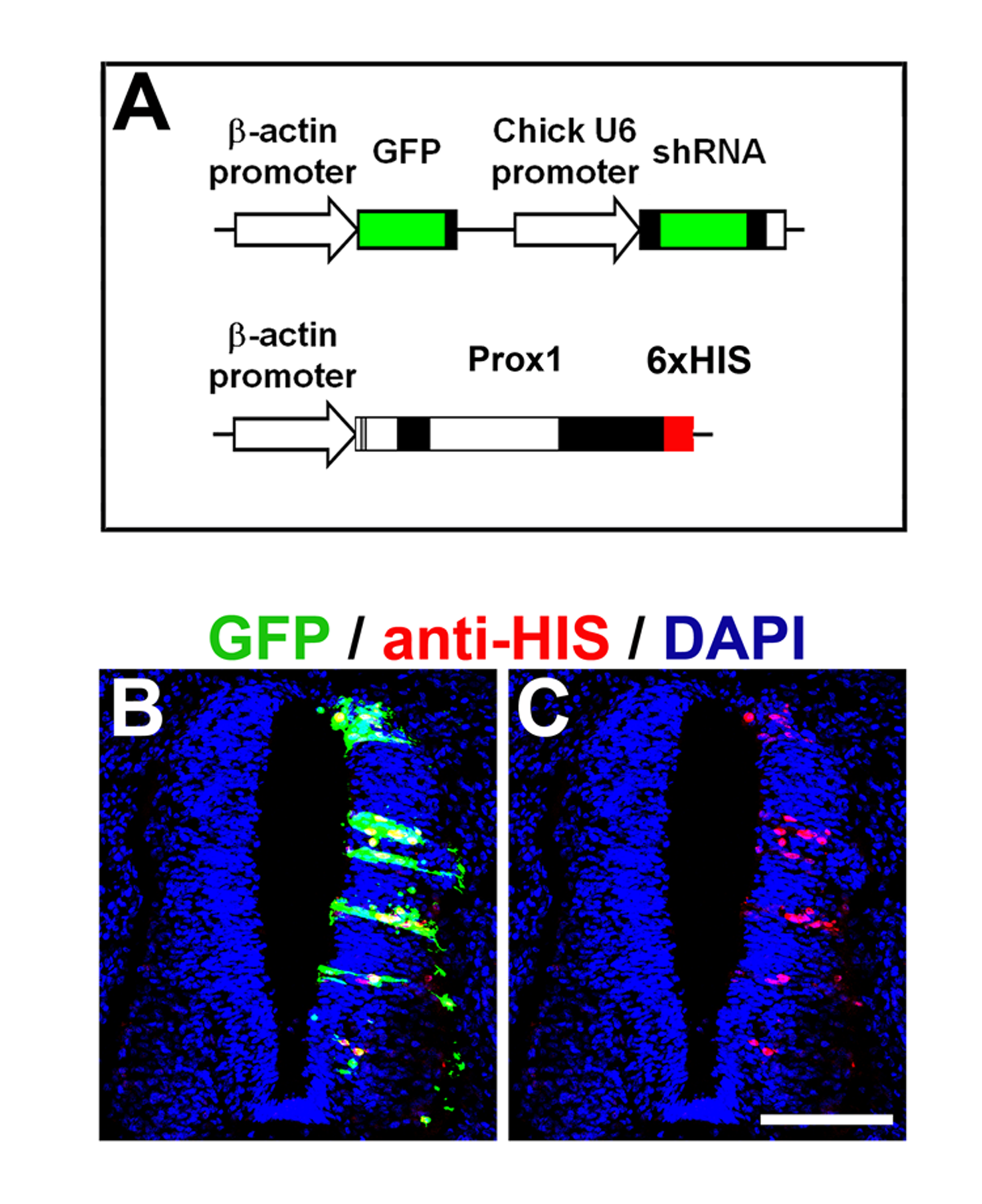

Supplement: Figure S9 — Rescue of the shRNA-Prox1 mediated phenotypes by murine Prox1. (A) Schematic representation of the shProx1 and murine Prox1 expression vectors. Note that shProx1 can be detected with GFP and murine Prox1 is tagged with 6xHIS epitope, and thus can be detected with anti-HIS immunostaining. (B–C) Double GFP/HIS immunostaining 48 h after co-electroporation of shProx1 and murine Prox1 constructs. Note that murine Prox1 is distributed in the same cells as the shProx1 construct. Scale bar: 100 µm. (4.11 MB TIF) [file pbio.1000565.s009.tif]

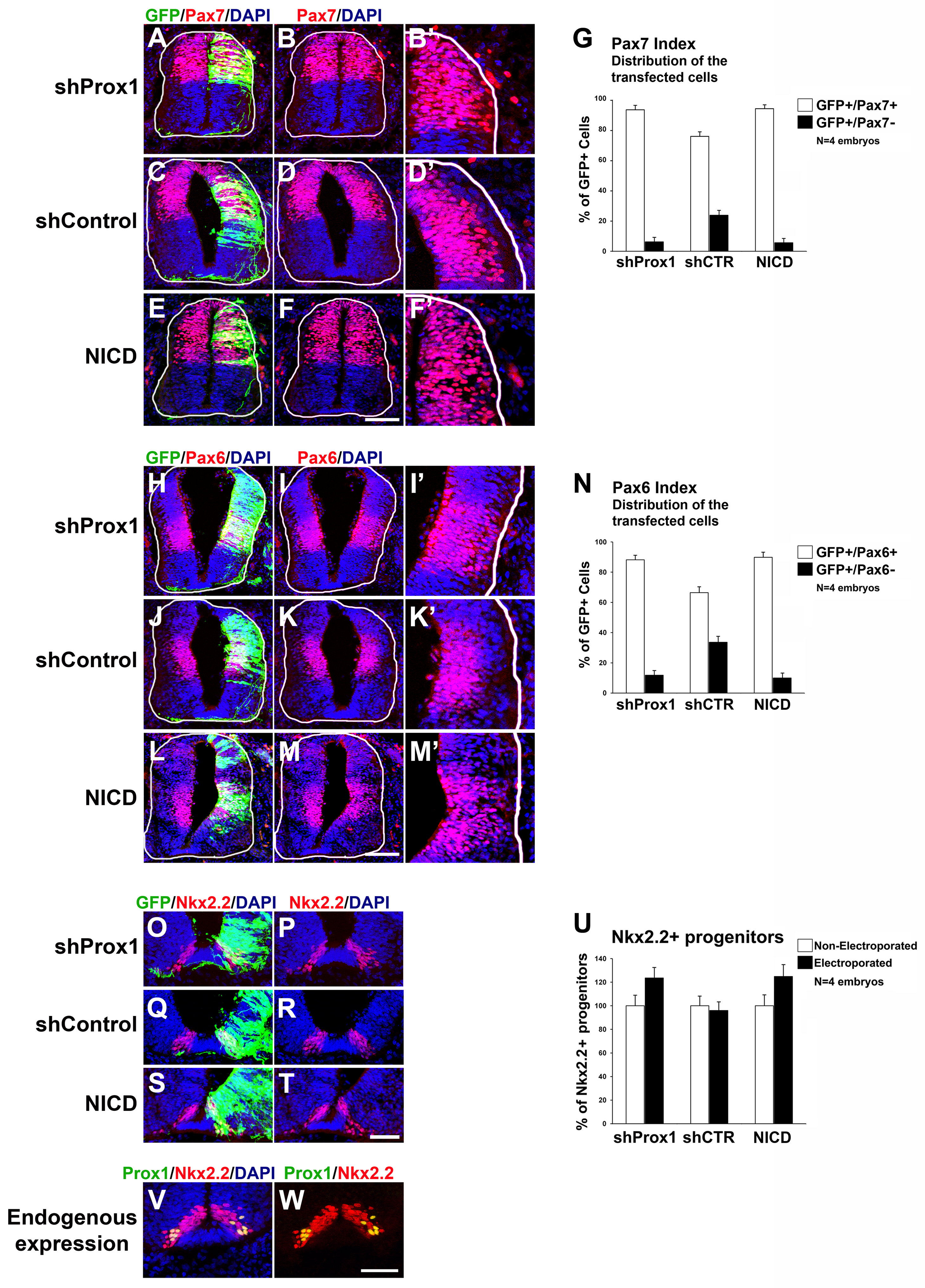

Supplement: Figure S10 — Prox1 is necessary for the suppression of Pax7, Pax6, and Nkx2.2 expression in the chick embryonic spinal cord. (A–F) Double GFP/Pax7 immunostainings 48 h a.e. with shProx1 (A–B), shControl (C–D), or NICD/GFP (E–F). The white lines indicate the outline of the spinal cord. (B'), (D'), and (F') micrographs are larger magnifications of the electroporated area in (B), (D), and (F), respectively. Scale bar: 75 µm. (G) Quantitative analysis of the number of transfected cells (GFP+) that are Pax7+ (white columns) or Pax7- (black columns). The data are presented as % of the total number of transfected cells (GFP+), n = 4 embryos. For GFP+/Pax7−, shProx1 versus shControl, p<0.01; shControl versus NICD/GFP, p<0.01; shProx1 versus NICD/GFP, p>0.1. (H–M) Double GFP/Pax6 immunostainings 48 h a.e. with shProx1 (H–I), shControl (J–K), or NICD/GFP (L–L'). The white lines indicate the outline of the spinal cord. (I'), (K'), and (M') micrographs are larger magnifications of the electroporated area in (I), (K), and (M), respectively. Scale bar: 75 µm. (N) Quantitative analysis of the number of transfected cells (GFP+) that are Pax6+ (white columns) or Pax6− (black columns). The data are presented as % of the total number of transfected cells (GFP+), n = 4 embryos. For GFP+/Pax6−, shProx1 versus shControl, p<0.01; shControl versus NICD/GFP, p<0.01; shProx1 versus NICD/GFP, p>0.1. (O–T) Double GFP/Nkx2.2 immunostainings 48 h a.e. with shProx1 (O–P), shControl (Q–R), or NICD/GFP (S–T). Scale bar: 50 µm. (U) Quantitative analysis of the number of Nkx2.2+ cells presented in (O–T). The data are presented as % of non-electroporated side of the spinal cord. For shProx1 versus shControl, p<0.05; shControl versus NICD/GFP, p<0.05; shProx1 versus NICD/GFP, p>0.1, n = 4 embryos. All cases referred to the electroporated side. (V–W) Endogenous Prox1 is expressed in a subset of Nkx2.2+ cells in the ventral spinal cord. Double Prox1/Nkx2.2 immunostainings in transverse sections of wild type embryonic [file pbio.1000565.s010.tif]

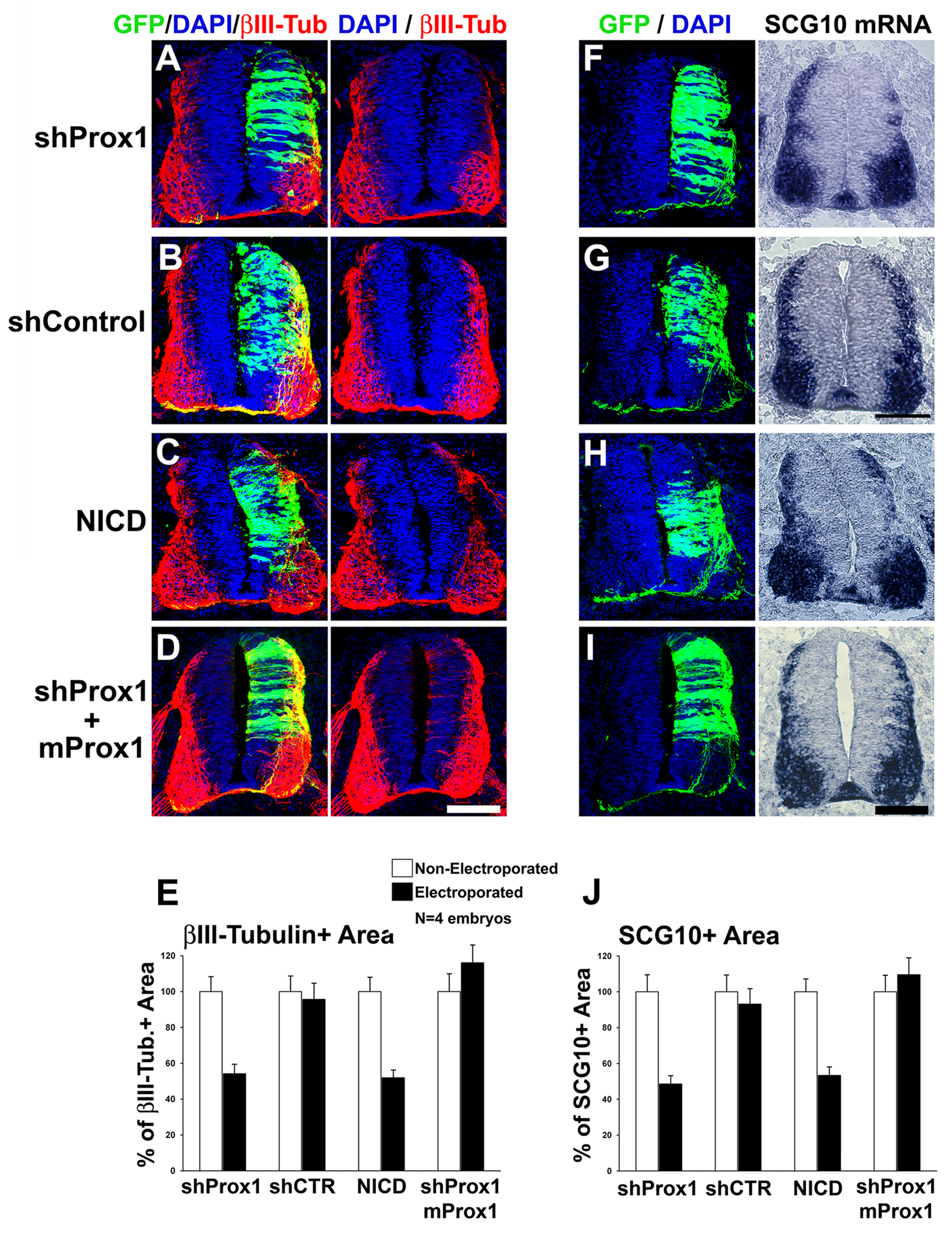

Supplement: Figure S11 — shRNA-mediated inhibition of Prox1 expression in chick spinal cord impairs neurogenesis. (A–D) Double GFP/βIII-tubulin immunostainings 48 h a.e. with shProx1 (A), shControl (B), NICD+GFP (C), or shProx1+mProx1 (D). Scale bar: 100 µm. (E) Quantitative analysis of the βIII-tubulin+ areas presented in (A–D) using the ImageJ software. The data are presented as % of non-electroporated side of the spinal cord. shProx1 versus shControl, p<0.01; shControl versus NICD, p<0.01; shProx1 versus NICD, p>0.1; shProx1 versus shProx1+mProx1, p<0.01, n = 4 embryos. All cases referred to the electroporated side. (F–I) GFP/DAPI stainings and in situ hybridization for SCG10 in consecutive sections 48 h a.e. with shProx1 (F), shControl (G), NICD+GFP (H), or shProx1+mProx1 (I). Scale bar: 100 µm. (J) Quantitative analysis of the SCG10+ area presented in (F–I) using the ImageJ software. The data are presented as % of non-electroporated side of the spinal cord. shProx1 versus shControl, p<0.01; shControl versus NICD, p<0.01; shProx1 versus NICD, p>0.1; shProx1 versus shProx1+mProx1, p<0.01, n = 4 embryos. All cases referred to the electroporated side. (8.59 MB TIF) [file pbio.1000565.s011.tif]

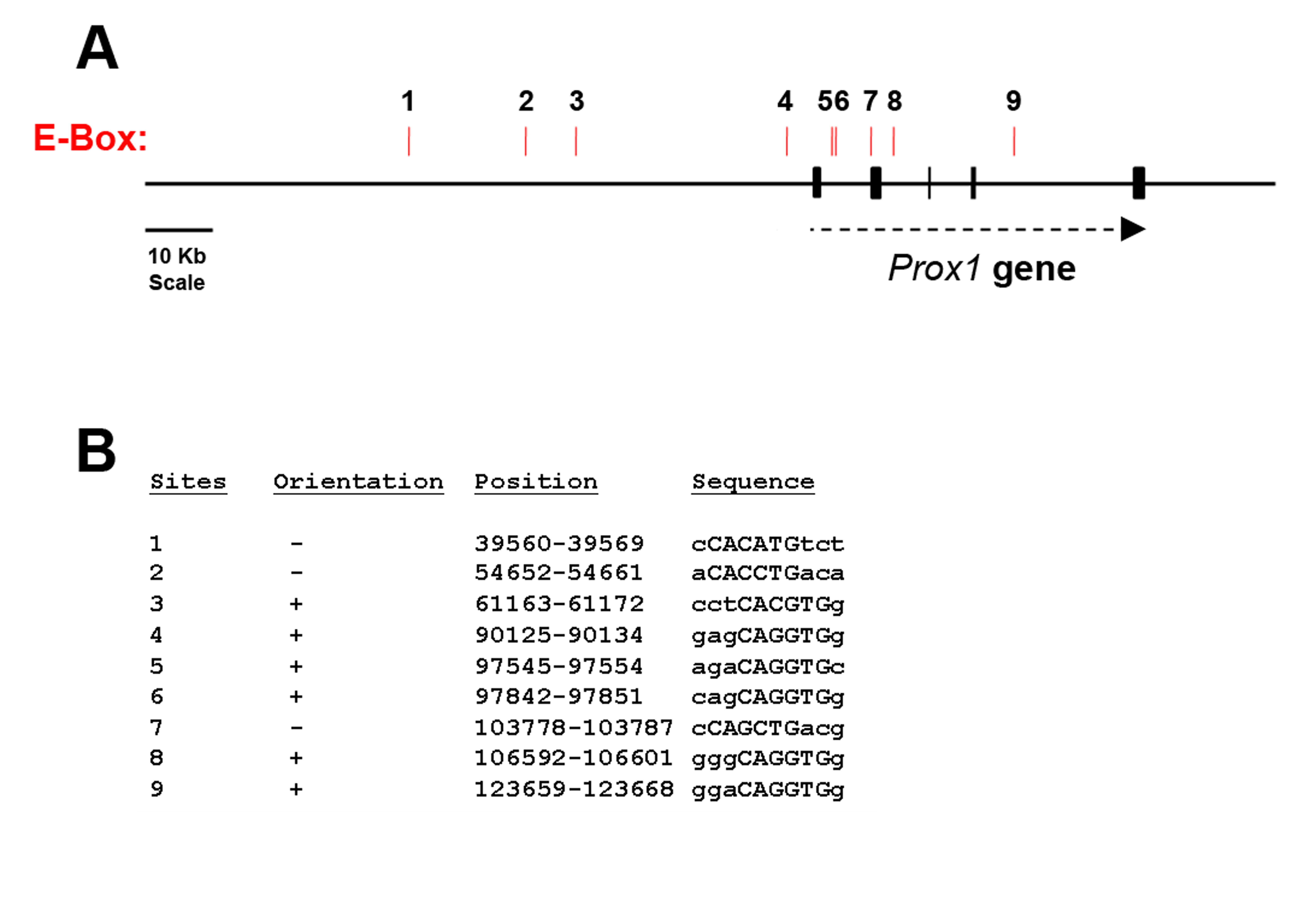

Supplement: Figure S12 — Schematic representation of the conserved binding sites (E-boxes) for proneural proteins on the mouse Prox1 gene locus. (A–B) 168 kb of the mouse Prox1 gene locus (100 kb upstream and 20 kb downstream of the Prox1 gene, chromosome 1: from 192094506 to 191926560) were aligned with the corresponding area of the human genome and the conserved binding sites for proneural proteins were identified as indicated (red lines). This analysis was performed with the ECR Browser software tool, freely available at http://ecrbrowser.dcode.org. Nine conserved putative binding sites were identified, based on the E-box consensus sequence, as previously published (CANNTG) [5],[53]. Detailed description of the orientation, position, and sequence of each site is indicated in (B). (1.86 MB TIF) [file pbio.1000565.s012.tif]
